# Graph-based modeling of optical system enables adaptive optics on dynamic samples with self-calibration

**DOI:** 10.1016/j.isci.2026.116769

**Published:** 2026-07-14

**Authors:** Eun-Seo Cho, Joon Park, Hyungwon Jin, Yoonjae Chung, Minho Eom, Hyejin Shin, Jae-Byum Chang, Jung-Hoon Park, Young-Gyu Yoon

**Affiliations:** 1 School of Electrical Engineering, KAIST, Daejeon, Republic of Korea; 2 Department of Biomedical Engineering, Ulsan National Institute of Science and Technology (UNIST), Ulsan, Republic of Korea; 3 Department of Materials Science and Engineering, KAIST, Daejeon, Republic of Korea; 4 Department of Semiconductor System Engineering, KAIST, Daejeon, Republic of Korea; 5 KAIST Institute for Human Augmentation Convergence, Daejeon, Republic of Korea

**Keywords:** adaptive optics, graph-based modeling, phase diversity, wavefront sensing

## Abstract

Sensor less adaptive optics offers significant advantages over hardware-based wavefront sensing but faces persistent challenges: Its performance degrades when idealized models fail to capture system imperfections, it is largely restricted to spatially invariant aberrations, and it cannot accommodate dynamic biological samples due to static-object assumptions. Here we present graph-modeling and phase-diversity-based computational adaptive optics with self-calibration (GRAPHYCS), a differentiable graph-based modeling framework that addresses all three limitations. GRAPHYCS automatically self-calibrates to correct system-specific non-idealities, enables spatially variant wavefront sensing across extended fields of view by modeling local aberrations, and supports dynamic live-sample imaging where conventional computational methods fail. In simulations, GRAPHYCS achieves up to a 9-fold improvement in wavefront sensing accuracy compared to analytic phase diversity under system non-idealities. In real microscopy experiments, it consistently outperforms phase-diversity-based methods compared in this study. Furthermore, in live zebrafish brain imaging, GRAPHYCS enables simultaneous wavefront sensing and neuronal activity detection—an application beyond the reach of existing approaches without additional hardware complexity.

## Highlights

• Differentiable graph-based modeling framework for computational adaptive optics
• Self-calibration enables accurate system- and sample-induced aberration correction
• Spatially varying aberration sensing across large fields of view
• Aberration correction in light-sheet microscopy of dynamic live biological samples

## Graphical abstract

**Figure:**
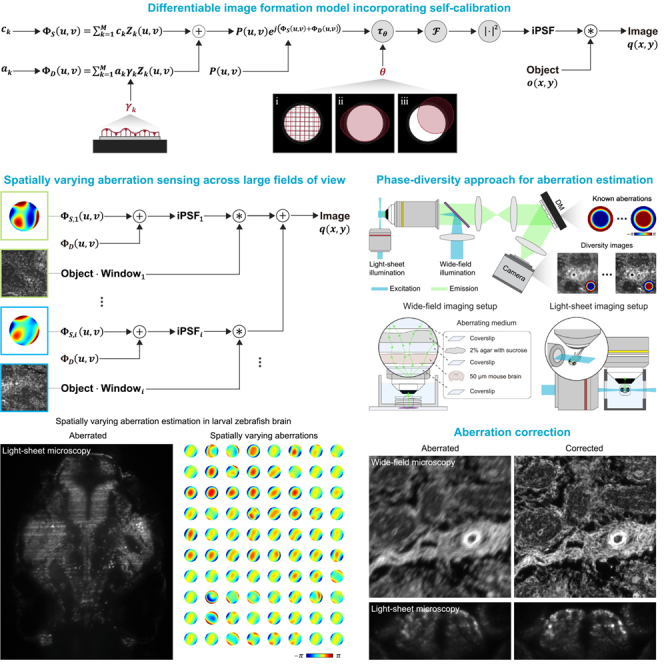

## Teaser

Applied sciences; Optical Signal Processing

## Introduction

Fluorescence microscopy has become an invaluable tool for visualizing biological structures and processes at cellular and subcellular levels.^1^^,^^2^^,^^3^^,^^4^^,^^5^^,^^6^^,^^7^^,^^8^^,^^9^^,^^10^ However, its performance is fundamentally constrained by optical aberrations that arise from refractive index heterogeneities in biological samples, optical misalignments, and imperfections in system components.^11^^,^^12^^,^^13^ These wavefront aberrations prevent diffraction-limited resolution, causing significant degradation in image quality, contrast, and signal-to-noise ratio, ultimately limiting our ability to observe fine structural details within living specimens.

The challenge of aberration correction becomes particularly acute when imaging across large fields of view, where heterogeneous refractive index distributions create spatially varying wavefront distortions. While high-resolution imaging has traditionally been limited to small regions where aberrations remain relatively uniform, many biological questions require observing extended areas—such as neural circuits spanning hundreds of micrometers or tissue sections exhibiting spatial organization at multiple scales. Achieving aberration correction across such extended regions represents a significant unmet need in biological imaging.

To overcome these limitations, adaptive optics (AOs) techniques have been developed to compensate for optical aberrations by measuring and correcting wavefront distortions.^11^^,^^12^^,^^13^^,^^14^^,^^15^^,^^16^^,^^17^^,^^18^^,^^19^^,^^20^^,^^21^^,^^22^ Originally developed for astronomical applications to mitigate atmospheric turbulence effects, AO has since been successfully adapted for biological imaging applications. Conventional AO methods in microscopy typically employ wavefront sensors, such as the Shack-Hartmann (SH) sensor, for direct measurement of wavefront distortions, and corrective elements such as deformable mirrors (DMs) or spatial light modulators (SLMs) to compensate for these aberrations.

While effective, sensor-based AO approaches face several challenges in microscopy applications, particularly for large field of view imaging. They require additional optical hardware that increases system complexity, cost, and introduces potential alignment errors. Furthermore, they often depend on the presence of a guide star or point-like reference source within the field of view, which is not naturally present in biological samples and must be artificially introduced through techniques such as two-photon excitation^15^^,^^16^ or embedding of fluorescent beads.^17^^,^^18^ When imaging large fields of view, multiple guide stars must be distributed throughout the sample, further complicating implementation. These requirements limit the practical application of sensor-based AO in many biological imaging scenarios.

Computational or sensor less AO methods offer a promising alternative by eliminating the need for dedicated wavefront sensing hardware and guide stars.^11^^,^^12^^,^^13^^,^^23^^,^^24^^,^^25^^,^^26^^,^^27^^,^^28^^,^^29^^,^^30^ Instead, these approaches estimate wavefront aberrations directly from image data using computational algorithms. For example, reflection-matrix-based approaches^29^ enable computational conjugate AO for correcting complex aberrations in deep tissue imaging, but require acquisition of complex reflection-matrix measurements. Learning-based frameworks^30^ have also been explored to infer aberrations directly from image measurements, although their performance depends on the availability of training data. Among various sensor less AO techniques, phase-diversity (PD) based wavefront sensing stands out as a particularly promising approach.^29^^,^^30^^,^^31^^,^^32^^,^^33^^,^^34^^,^^35^^,^^36^^,^^37^^,^^38^^,^^39^ PD was originally developed over 30 years ago in astronomy, utilizing additional known phase aberrations and the Gerchberg-Saxton algorithm to estimate unknown aberrations and the underlying object structure.

In 2024, the concept of PD was successfully demonstrated in fluorescence microscopy through an analytic approach that employs numerical optimization based on fixed-form equations, which we refer to as analytic PD.^38^ This recent adaptation has shown success for aberration estimation under idealized conditions in microscopy settings.

In practical optical systems, subtle geometric misalignments or hardware variations can create discrepancies between the computational model and the physical system. The analytic PD wavefront sensing relies on numerical optimization based on fixed-form equations that presume an idealized optical configuration. This formulation, while mathematically elegant, can face challenges when applied to real-world systems where the actual conditions diverge from the idealized model. Under such circumstances, the estimated aberrations may not fully capture the true phase distortions, potentially leading to incomplete aberration correction and limiting the achievable image quality improvement.

Furthermore, computational AO approaches based on simple optical models, including analytic PD, assume spatially invariant aberrations across the entire field of view.^27^^,^^28^^,^^38^ While this assumption holds for small imaging regions (typically <100 μm^2^), it becomes invalid when imaging larger areas of biological samples with heterogeneous refractive index distributions that cause spatially varying aberrations. The limited field of view in current AO approaches presents a significant practical constraint for many biological imaging applications that require visualization of extended cellular networks, tissue architectures, or organ-level structures.

In addition, existing computational AO approaches face an additional fundamental limitation—they assume the imaged object remains static throughout the PD acquisition sequence. This assumption becomes problematic when imaging live biological specimens where specimen drift and dynamic biological processes such as neuronal activity occur naturally during the time required to acquire multiple diversity images.

To address these challenges, we propose GRAPHYCS (graph-modeling and phase-diversity-based computational AO with self-calibration), a computational AO framework that bridges the gap between computational models and real optical systems. GRAPHYCS represents the image formation process as a differentiable computational graph with learnable parameters that can adapt to system-specific characteristics.

This differentiable graph formulation enables three key extensions that overcome fundamental limitations of existing computational methods. First, it incorporates learnable calibration parameters that automatically compensate for system-specific non-idealities—capabilities that fixed-form analytic methods cannot accommodate. Second, it extends to spatially variant modeling, enabling aberration correction across large fields of view (>1 mm^2^) where conventional methods assume spatial invariance. Third, it can incorporate temporal object dynamics for live biological imaging, where conventional PD methods fail due to static object assumptions. These extensions are enabled by the flexibility of differentiable graphs, which allow simultaneous optimization of imaging parameters.

We validate GRAPHYCS through simulations and experimental demonstrations across three key capabilities. Our simulations demonstrate that GRAPHYCS accurately estimates and corrects optical aberrations under system non-idealities, achieving up to a 9-fold improvement in wavefront sensing accuracy compared to analytic PD that assumes idealized conditions. In experimental validation with biological specimens, we show that GRAPHYCS effectively compensates for both system- and sample-induced aberrations, with quantitative improvements in all metrics. We demonstrate GRAPHYCS’s ability to handle spatially varying aberrations across large fields of view exceeding 1 mm^2^, enabling aberration correction in samples with heterogeneous refractive index distributions. Additionally, we validate its capability to image dynamic samples through live larval zebrafish brain imaging with light-sheet microscopy, where GRAPHYCS successfully performs simultaneous aberration correction and dynamic activity detection—applications where conventional computational methods fail due to their static object assumptions.

## Results

### GRAPHYCS: graph-based computational adaptive optics with self-calibration

We developed GRAPHYCS around a core innovation—formulating optical imaging as a differentiable computational graph that enables automatic optimization of parameters constrained in conventional analytic methods. This approach addresses three critical limitations of existing computational wavefront sensing for AO. First, performance degradation in real systems due to model-hardware mismatches. Second, restriction to spatially invariant aberrations that limits field of view. Third, requirement for static samples that prevents live imaging applications. By treating system parameters, spatial variations, and temporal changes as learnable components within the computational graph, GRAPHYCS can simultaneously optimize aberration estimation, object estimation, and calibration parameters through unified gradient-based optimization. This flexibility distinguishes our approach from conventional methods assuming idealized conditions.

For the graph construction, we start by considering the incoherent image formation model in fluorescence microscopy, expressed as:

q(x,y)=o(x,y)∗h(x,y),

where ∗ denotes the convolution operation, *o*(*x*,*y*) represents the object, *q*(*x*,*y*) represents the image, *h*(*x*,*y*) represents the intensity point-spread function (iPSF) of the imaging system, and *x* and *y* denote the spatial coordinates on the image plane.

The iPSF *h*(*x*,*y*) is affected by the wavefront aberration Φ_*S*_(*u*,*v*), which can be caused by either the sample or the optical system itself, and the pupil function *P*(*u*,*v*), where *u* and *v* denote the spatial coordinates on the pupil plane. Using Fourier optics, the iPSF *h*(*x*,*y*) can be represented as:

$$h\left(x,y\right)=|F\left\{P\left(u,v\right)e^{j\Phi_{S}\left(u,v\right)}\right\}{|}_{f_{u}=\frac{x}{\lambda l},f_{v}=\frac{y}{\lambda l}}^{2}\text{,}$$

where, F{·} denotes a two-dimensional Fourier transform with respect to *u* and *v*, *f*_*u*_ and *f*_*v*_ denote the spatial frequencies in *u* and *v* dimensions, *λ* denotes the wavelength of emission light, and *l* denotes the focal length of the tube lens of the microscope.

Thus, the image formation model can be written as:

$$q\left(x,y\right)=o\left(x,y\right)*|F\left\{P\left(u,v\right)e^{j\Phi_{S}\left(u,v\right)}\right\}{|}_{f_{u}=\frac{x}{\lambda l},f_{v}=\frac{y}{\lambda l}}^{2}\text{.}$$

**Figure 1:**
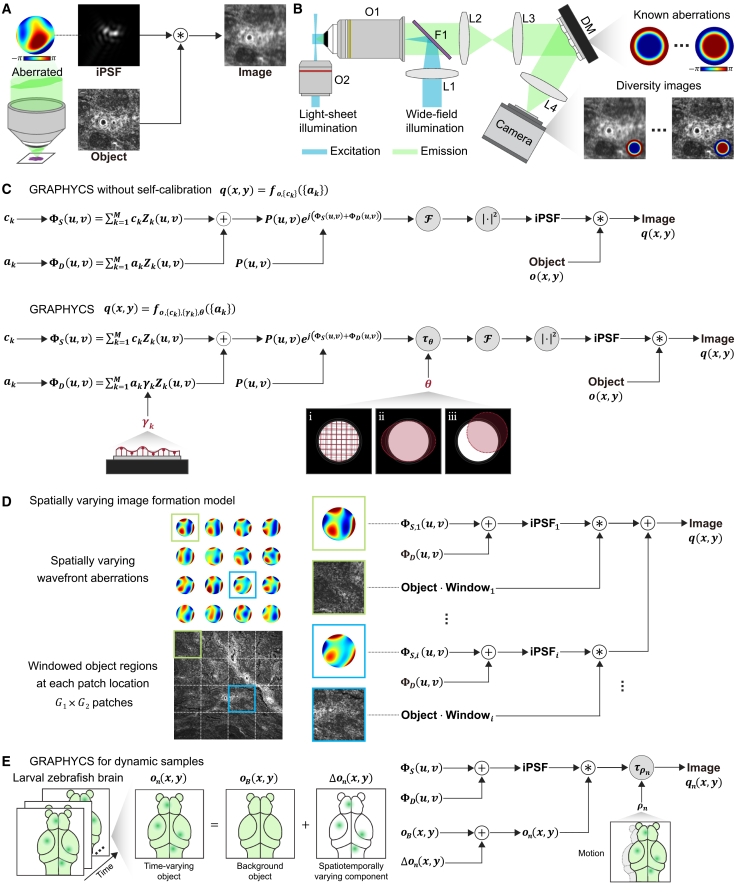
Differentiable graph-based modeling enables computational adaptive optics on dynamic samples with self-calibration and spatially varying aberration estimation (A) Schematic of the incoherent image formation model in fluorescence microscopy expressed as a computational graph. (B) Phase-diversity approach for aberration estimation. Schematic of our adaptive optics system integrated with a deformable mirror (DM) placed conjugate to the pupil plane of the objective, enabling the acquisition of multiple images with deliberately introduced known aberrations. (C) Schematic of the differentiable image formation model incorporating self-calibration. GRAPHYCS without self-calibration assumed an idealized optical system (top), whereas GRAPHYCS (bottom) included learnable parameters for modeling optical non-idealities. (D) Schematic of image formation process incorporating spatially variant convolution by dividing the field of view into *G*_1_×*G*_2_ patches. (E) Schematic of GRAPHYCS for dynamic samples that incorporates a stationary background object, temporally varying component, and motion.

This model consists of only multiplication, convolution, exponential function, and Fourier transform operations, all of which are differentiable with respect to both *o* and Φ_*S*_. Therefore, both ∇_*o*_*q*(*x*,*y*) and $\nabla_{\Phi_{S}}q\left(x,y\right)$ can be computed using the chain rule, or in other words, through backpropagation along the computational graph (Figure 1A).

Since our observation *I*(*x*,*y*)—the measured image—is supposedly the same as *q*(*x*,*y*) aside from noise, we can define a loss function as the absolute error between them: L=|I(x,y)−q(x,y)|. This formulation opens the possibility of jointly optimizing both the object *o*(*x*,*y*) and Φ_*S*_(*u*,*v*) for a given observation *I*(*x*,*y*) by minimizing L. However, this approach presents an ill-posed problem as it becomes difficult to determine whether blurriness in the image originates from the object itself or from optical aberrations.

To address this ill-posedness, we employ a PD approach by deliberately introducing additional known wavefront aberrations using a DM placed on the Fourier plane of the imaging system. By capturing multiple images with different additional known aberrations, we can extract the necessary information from this set of images. The total aberration becomes the sum of the unknown system/sample aberration Φ_*S*_(*u*,*v*) and the deliberately introduced aberration Φ_*D*_(*u*,*v*), resulting in the modified image formation model:

$$q\left(x,y\right)=o\left(x,y\right)*|F\left\{P\left(u,v\right)e^{j\left(\Phi_{S}\left(u,v\right)+\Phi_{D}\left(u,v\right)\right)}\right\}{|}_{f_{u}=\frac{x}{\lambda l},f_{v}=\frac{y}{\lambda l}}^{2}\text{.}$$

To make the optimization process more robust, we parameterize both Φ_*S*_(*u*,*v*) and Φ_*D*_(*u*,*v*) using Zernike polynomials, which provide an orthogonal basis for representing wavefront aberrations:

$$\Phi_{S}\left(u,v\right)=\sum_{k=1}^{M}c_{k}Z_{k}\left(u,v\right),\Phi_{D}\left(u,v\right)=\sum_{k=1}^{M}a_{k}Z_{k}\left(u,v\right)\text{,}$$

where *Z*_*k*_ is the *k*th order Zernike polynomial, *c*_*k*_ and *a*_*k*_ are the corresponding coefficients, and *M* is the maximum order of Zernike polynomial that we use for approximating the system- and sample-induced aberration. By incorporating this Zernike representation, we obtain the following:

$$q\left(x,y\right)=f_{o,\left\{c_{k}\right\}}\left(\left\{a_{k}\right\}\right)=o\left(x,y\right)*|F\{P\left(u,v\right)e^{j\left(\sum_{k=1}^{M}c_{k}Z_{k}\left(u,v\right)+\sum_{k=1}^{M}a_{k}Z_{k}\left(u,v\right)\right)}\}{|}_{f_{u}=\frac{x}{\lambda l},f_{v}=\frac{y}{\lambda l}}^{2}\text{.}$$

This formulation treats *o* and *c*_*k*_ as the parameters of the function *f*, *a*_*k*_ as the input, and *q* as the output. By acquiring a set of images {*I*_*n*_} corresponding to different known aberrations specified by $\left\{{\left\{a_{k}\right\}}_{n}\right\}$, we essentially collect input-output pairs that characterize this function as illustrated in Figure 1B. We can then define a loss function as the sum of the absolute error across all image pairs in the image and the image spectral domains: $L=\sum_{n=1}^{N}\left|I_{n}\left(x,y\right)-q_{n}\left(x,y\right)\right|+\alpha \left|{F\{I}_{n}\left(x,y\right)\}-{F\{q}_{n}\left(x,y\right)\}\right|$, where *α* is a hyperparameter that determines the ratio of the two loss terms. The optimal values for *o* and *c*_*k*_ can be determined using gradient-based optimization with $\nabla_{o}L$ and $\nabla_{c_{k}}L$ computed through backpropagation (Figure 1C).

While the underlying philosophy of using additional images with known aberrations remains consistent with previous PD methods,^31^^,^^32^^,^^33^^,^^34^^,^^35^^,^^36^^,^^37^^,^^38^^,^^39^ our graph-based approach offers significant advantages in addressing real-world optical non-idealities. In practice, several factors can cause mismatches between the computational model and the physical optical system, including limited alignment accuracy, coordinate discrepancies between the DM control software and the model, and inconsistencies between the specified and actual mirror deformations.^38^^,^^40^^,^^41^

The flexibility of our graph-based formulation allows us to extend the image formation model to include these non-idealities (Figure 1C); Our model can incorporate calibration parameters that can be automatically learned during the optimization process:

$$q\left(x,y\right)=f_{o,\left\{c_{k}\right\},\left\{\gamma_{k}\right\},\theta }\left(\left\{a_{k}\right\}\right)=o\left(x,y\right)*|F\left\{\tau_{\theta }\left\{P\left(u,v\right)e^{j\left(\sum_{k=1}^{M}c_{k}Z_{k}\left(u,v\right)+\sum_{k=1}^{M}a_{k}\gamma_{k}Z_{k}\left(u,v\right)\right)}\right\}\right\}{|}_{f_{u}=\frac{x}{\lambda l},f_{v}=\frac{y}{\lambda l}}^{2}\text{.}$$

Here, {*γ*_*k*_} represents DM stroke calibration parameters for each Zernike mode, and *τ*_*θ*_{·} denotes an affine transformation parameterized by *θ*. This affine transformation elegantly captures various system mismatches including coordinate discrepancies, beam size variations on the Fourier plane, and misalignments between the beam and the DM center (Figure 1C). The key aspect of our approach is that the entire model remains differentiable with respect to all parameters of interest: *o*, {*c*_*k*_}, {*γ*_*k*_}, and *θ*. This enables simultaneous self-calibration, wavefront aberration estimation, and object estimation through a unified gradient-based optimization framework.

The graph architecture naturally extends to accommodate spatial heterogeneity across the imaging field. We implemented GRAPHYCS with a spatially variant image formation model that accounts for local wavefront distortions (Figure 1D). The image formation models that assume spatially invariant aberrations across the entire field of view become inadequate when imaging samples with heterogeneous refractive indices, where aberrations can vary significantly across different regions of the imaging area.

Our spatially varying model divides the image into a set of *G*_1_×*G*_2_ patches (Figure 1D). For each patch, we define a local wavefront aberrations Φ_*S*,*i*_(*u*,*v*)parameterized by position-dependent Zernike coefficients:

$$\Phi_{S,i}\left(u,v\right)=\sum_{k=1}^{M}c_{k,i}Z_{k}\left(u,v\right)\text{,}$$

where *c*_*k*,*i*_ represents the coefficient at the *i*-th patch location. This formulation allows us to model spatially varying wavefront distortions that commonly occur in samples with inhomogeneous refractive indices.

The image formation process for this spatially varying model can be expressed as a weighted sum of local convolutions between windowed object regions and spatially varying iPSFs:

$$q\left(x,y\right)=\sum_{i}\left(o\left(x,y\right)\cdot w_{i}\left(x,y\right)\right)*h_{i}\left(x,y\right)\text{,}$$

where *w*_*i*_ represents a window function at the *i*-th region, {·}denotes element-wise multiplication, and *h*_*i*_ represents the spatially varying iPSF with *i*-th region of the imaging system. Incorporating these elements into our graph-based model, we obtain:

$$q\left(x,y\right)=f_{o,\left\{c_{k,i}\right\},\left\{\gamma_{k}\right\},\theta }\left(\left\{a_{k}\right\}\right)=\sum_{i}\left(o\left(x,y\right)\cdot w_{i}\left(x,y\right)\right)*|F\left\{\tau_{\theta }\left\{P\left(u,v\right)e^{j\left(\sum_{k=1}^{M}c_{k,i}Z_{k}\left(u,v\right)+\sum_{k=1}^{M}a_{k}\gamma_{k}Z_{k}\left(u,v\right)\right)}\right\}\right\}{|}_{f_{u}=\frac{x}{\lambda l},f_{v}=\frac{y}{\lambda l}}^{2}\text{.}$$

To ensure smooth variation of aberrations across the field of view, we decompose the aberration coefficients into a common mode coefficient ${c_{k}}^{cm}$ that affects the entire field of view and a spatially varying aberration component Δ*c*_*k*,*i*_:

$$c_{k,i}={c_{k}}^{cm}+\Delta c_{k,i}\text{.}$$

To handle temporal object changes during PD acquisition, we decompose the object into a background object *o*_*B*_(*x*,*y*) that represents a stationary baseline component and a temporally varying component Δ*o*_*n*_(*x*,*y*):

$$o_{n}\left(x,y\right)=o_{B}\left(x,y\right)+\Delta o_{n}\left(x,y\right)\text{,}$$

and account for motion induced by specimen drift using an affine transformation $\tau_{\rho_{n}}\left\{\cdot \right\}$ applied prior to image formation (Figure 1E). Integrating these elements into our graph-based formulation, the image formation model can be represented as:

$$q_{n}\left(x,y\right)=f_{o_{B},\Delta o_{n},\left\{c_{k}\right\},\left\{\gamma_{k}\right\},\theta ,\rho_{n}}\left(\left\{a_{k}\right\}\right)$$

$$=\tau_{\rho_{n}}{\left\{\left\{o_{B}\left(x,y\right)+\Delta o_{n}\left(x,y\right)\right\}*{\left|F\left\{\tau_{\theta }\left\{P\left(u,v\right)e^{j\left(\sum_{k=1}^{M}c_{k}Z_{k}\left(u,v\right)+\sum_{k=1}^{M}a_{k}\gamma_{k}Z_{k}\left(u,v\right)\right)}\right\}\right\}\right|}^{2}\right\}}_{f_{u}=\frac{x}{\lambda l},f_{v}=\frac{y}{\lambda l}}$$

### Performance validation on simulated data

For quantitative evaluations of GRAPHYCS’s performance, we validated its performance on synthetic wide-field microscopy data, which were generated using commercial optical simulation software (Zemax Optic Studio). First, we designed an optical model of our wide-field AO setup as shown in Figure S1.

To assess how well the algorithm estimates unknown optical aberrations and object structure when faced with discrepancies between the computational model and physical imaging system, we created two distinct datasets: The first dataset contained ideal simulations without system imperfections, while the second incorporated realistic system imperfections. These non-idealities were simulated by introducing controlled misalignments to the DM: a lateral shift of −2.0 mm in *x* and +1.0 mm in *y* directions, representing displacement between the DM center and the system’s optical axis, and a 1.0-degree additional tilt relative to the nominal 7.5-degree incident angle, simulating misalignment in the emission light path relative to the DM surface. For the dataset simulated with system non-idealities, we generated synthetic datasets with different underlying wavefront aberrations under the same non-ideal conditions (*n* = 15). This experimental design allowed us to assess performance under conditions that closely approximate real-world optical system limitations.

**Figure 2:**
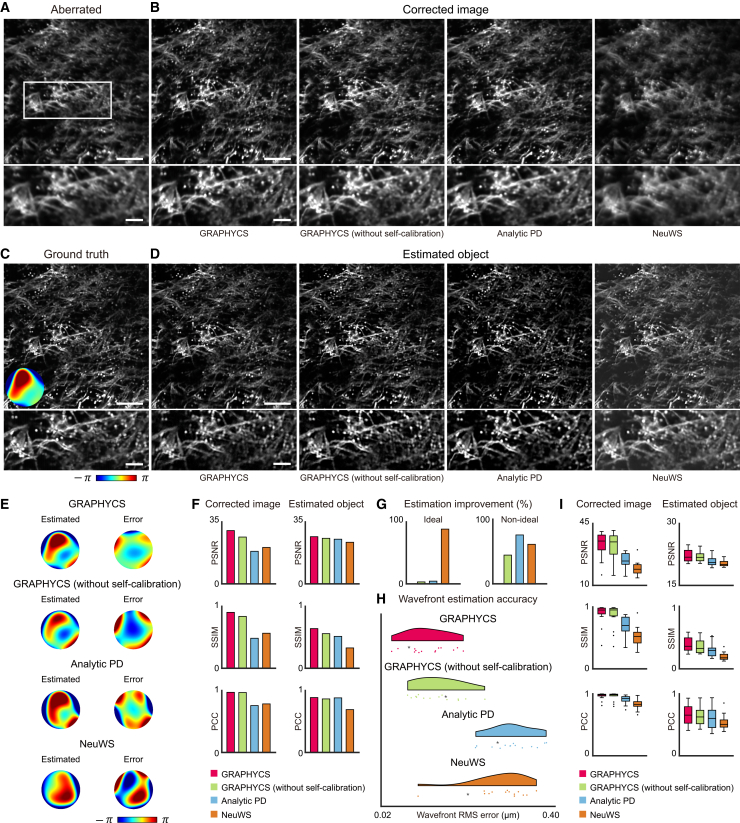
Performance validation on simulated data (A) Aberrated image simulated with system non-idealities. Scale bars, 50 μm. A magnified view of the boxed region is presented below. Scale bars, 20 μm. (B) Aberration-corrected images of GRAPHYCS, GRAPHYCS without self-calibration, analytic PD, and NeuWS (from left to right). Scale bars, 50 μm. A magnified view of the boxed region in (A) is presented below. Scale bars, 20 μm. (C) Ground truth object image along with ground truth wavefront aberrations. Scale bars, 50 μm. A magnified view of the boxed region in (A) is presented below. Scale bars, 20 μm. (D) Estimated object images of GRAPHYCS, GRAPHYCS without self-calibration, analytic PD, and NeuWS (from left to right). Scale bars, 50 μm. A magnified view of the boxed region in (A) is presented below. Scale bars, 20 μm. (E) Estimated wavefront aberrations (left column) and corresponding wavefront error maps (right column) from GRAPHYCS, GRAPHYCS without self-calibration, analytic PD, and NeuWS (top to bottom). (F) Quantitative comparison of image quality metrics (PSNR, SSIM, and PCC) for the aberration-corrected images (left) and the estimated object images (right) under non-ideal conditions. (G) Relative wavefront aberration estimation improvement (%) under ideal (left) and non-ideal (right) conditions. (H) Raincloud-style plots of wavefront aberration estimation accuracy for GRAPHYCS, GRAPHYCS without self-calibration, analytic PD, and NeuWS (top to bottom) across simulations under same non-ideal conditions (*n* = 15). The distributions show wavefront RMS error across the simulations, the dots represent the individual simulation results, and the gray stars indicate the result shown in (E). (I) Box-and-whisker plots show image quality metrics (PSNR, SSIM, and PCC) for the aberration-corrected images (left) and the estimated object images (right) across the simulations under the same non-ideal conditions (*n* = 15). Center lines indicate medians, boxes indicate the 25th–75th percentiles, and whiskers extend to the most extreme values within 1.5 times the interquartile range.

We used a confocal microscope image of a *Penicillium* as the ground truth object data with the same pixel size of our system. Aberrations were introduced using a linear combination of 12 Zernike modes (from primary astigmatism to tetrafoil, excluding piston, tip, and tilt) with randomized coefficients (Figure 2A; Figure S2A). To introduce additional known aberrations, we used defocus *Z*_5_as the applied phase at the DM plane, generating 11 diversity images by varying the *Z*_5_Zernike coefficient value in equal steps (for a total of 12 images, including the initial aberrated image). Poisson and Gaussian noise were added to the generated diversity images. Further details can be found in the STAR Methods section.

We compared four methods on the synthetic datasets: (1) GRAPHYCS, (2) GRAPHYCS without self-calibration, (3) analytic PD,^38^ and (4) NeuWS.^39^ For method (2), we disabled the self-calibration component by freezing the calibration parameters, thereby constraining the model to assume the idealized optical system, similar to analytic PD. We evaluated the performance of each method by comparing the estimated wavefront aberrations and estimated object images to ground truth data. Aberration estimation accuracy was quantified by calculating a root-mean-square (RMS) wavefront distortion (or wavefront RMS error) between the estimated and ground truth wavefront aberrations. To evaluate object estimation performance, we compared the estimated object images to the ground truth image using the peak signal-to-noise ratio (PSNR), structural similarity index measure (SSIM), and Pearson’s correlation coefficient (PCC). In addition, we assessed aberration correction performance using the same metrics (PSNR, SSIM, and PCC) by comparing the aberration-corrected images to the ideal, aberration-free image simulated with the optical simulation software.

Under ideal conditions, GRAPHYCS, GRAPHYCS without self-calibration, and analytic PD demonstrated effective recovery of structural details in the aberration-corrected images (Figure S2B). In contrast, NeuWS showed degraded aberration correction performance. This performance degradation resulted from the mismatch between the PD acquisition protocol used in this study and the original NeuWS configuration, which was designed for at least 100 images with random phase modulations. NeuWS evaluated under its original configuration achieved low wavefront RMS error consistent with published results (Figure S3; Table S1). The estimated object images obtained from all methods showed accurate recovery of structural features, closely matching the ground truth image under ideal conditions (Figures S2C and S2D). In the presence of system imperfections, the result of aberration correction using GRAPHYCS showed improved performance compared to the other methods. Structural details that remained blurred in the aberration-corrected images from GRAPHYCS without self-calibration, analytic PD, and NeuWS were better resolved when using GRAPHYCS (Figure 2B). The effectiveness of our method with self-calibration was further supported by additional simulations (Figures S4–S6). For object estimation performance, GRAPHYCS also demonstrated improvements over the other methods under non-ideal conditions. It accurately recovered fine structural details in the estimated object image, closely matching the ground truth image, which was degraded in the results from GRAPHYCS without self-calibration, analytic PD, and NeuWS (Figures 2C and 2D). These qualitative observations were supported by quantitative metrics.

All methods except NeuWS demonstrated low wavefront error under ideal conditions, indicating accurate aberration estimation (Figure S2E). GRAPHYCS achieved the lowest wavefront RMS error of 0.0289 μm, which was lower than that of GRAPHYCS without self-calibration, analytic PD, and NeuWS (Table 1). As the wavefront aberration estimation accuracy improved, GRAPHYCS achieved higher values across all metrics in the aberration-corrected image and the estimated object image under ideal conditions (Figure S2F). For the aberration-corrected images, GRAPHYCS showed improved image quality metrics, with a PSNR of 36.52 dB, SSIM of 0.9681, and PCC of 0.9862, compared to GRAPHYCS without self-calibration, analytic PD, and NeuWS. Similarly, for the estimated object images, GRAPHYCS demonstrated higher metric values compared to GRAPHYCS without self-calibration, analytic PD, and NeuWS (Table 1). The observed performance improvement with self-calibration, even under ideal conditions, can be attributed to inherent modeling discrepancies between our simulation and estimation approaches. Specifically, the synthetic data generation utilized OpticStudio for optical simulation, while our graph-based computational model employed Fourier optics principles. Consequently, even under ideal imaging conditions, subtle discrepancies exist between the computational model and the simulated optical system, providing an opportunity for the self-calibration mechanism to enhance performance by accounting for these differences.

**Table 1:** Quantitative comparison on simulated data under ideal conditions

Ideal conditions		Aberration-corrected image			Estimated object		
Method	Wavefront RMS error (μm)	PSNR (dB)	SSIM	PCC	PSNR (dB)	SSIM	PCC
GRAPHYCS	0.0289	36.52	0.9681	0.9862	27.01	0.6290	0.9616
GRAPHYCS (without self-calibration)	0.0304	35.13	0.9610	0.9853	26.27	0.5864	0.9542
Analytic PD	0.0308	33.72	0.9362	0.9691	26.50	0.6246	0.9636
NeuWS	0.3185	14.69	0.3056	0.6841	23.95	0.3336	0.8009

In the presence of system imperfections, GRAPHYCS demonstrated the lowest wavefront RMS error among all evaluated methods (Figure 2E). Both GRAPHYCS without self-calibration and analytic PD showed performance degradation under these non-ideal conditions, with wavefront RMS errors increasing notably compared to the ideal case (Figure S2E; Figure 2E). In contrast, GRAPHYCS maintained a low error of 0.0340 μm. This wavefront RMS error was lower than the RMS error of GRAPHYCS without self-calibration, analytic PD, and NeuWS (Table 2). GRAPHYCS achieved up to a 9-fold improvement in wavefront sensing accuracy compared to analytic PD across all simulations (Table S2). Moreover, this improvement in wavefront aberration estimation accuracy was reflected in both the aberration correction and object estimation performance (Figure 2F). In the aberration-corrected images, GRAPHYCS achieved higher values across all metrics, with a PSNR of 30.15 dB, SSIM of 0.9021, and PCC of 0.9728, compared to GRAPHYCS without self-calibration and analytic PD, and NeuWS. For object estimation performance, GRAPHYCS also demonstrated higher values than GRAPHYCS without self-calibration, analytic PD, and NeuWS (Table 2). The relative wavefront aberration estimation improvement of GRAPHYCS over the other methods under both ideal and non-ideal conditions further supported the effectiveness of self-calibration (Figure 2G). Furthermore, the simulations with different underlying aberrations under the same non-ideal conditions (*n* = 15) showed that GRAPHYCS generally achieved lower wavefront RMS error as well as improved aberration correction and object estimation performance compared to GRAPHYCS without self-calibration, analytic PD, and NeuWS (Figures 2H and 2I; Table 3).

**Table 2:** Quantitative comparison on simulated data under non-ideal conditions

Non-ideal conditions		Aberration-corrected image			Estimated object		
Method	Wavefront RMS error (μm)	PSNR (dB)	SSIM	PCC	PSNR (dB)	SSIM	PCC
GRAPHYCS	0.0340	30.15	0.9021	0.9728	26.71	0.6406	0.8881
GRAPHYCS (without self-calibration)	0.0637	26.49	0.8374	0.9699	25.79	0.5590	0.8663
Analytic PD	0.1553	18.43	0.4842	0.7589	25.27	0.5152	0.8832
NeuWS	0.0932	20.61	0.5658	0.7859	23.54	0.3286	0.6937

**Table 3:** Summary of quantitative comparison on simulated data under non-ideal conditions

Non-ideal conditions		Aberration-corrected image			Estimated object		
Method	Wavefront RMS error (μm)	PSNR (dB)	SSIM	PCC	PSNR (dB)	SSIM	PCC
	Method ± standard deviation						
GRAPHYCS	0.0494 ± 0.0183	33.00 ± 6.78	0.8811 ± 0.1647	0.9559 ± 0.0527	22.12 ± 1.41	0.3960 ± 0.1146	0.6578 ± 0.1676
GRAPHYCS (without self-calibration)	0.0597 ± 0.0252	31.92 ± 7.54	0.8529 ± 0.1938	0.9497 ± 0.0651	21.94 ± 1.27	0.3603 ± 0.1088	0.6359 ± 0.1586
Analytic PD	0.2115 ± 0.0794	24.20 ± 4.31	0.6992 ± 0.1547	0.8994 ± 0.0667	20.85 ± 1.15	0.2986 ± 0.1100	0.6033 ± 0.1660
NeuWS	0.1758 ± 0.0678	19.52 ± 3.97	0.5163 ± 0.1612	0.8203 ± 0.0798	20.27 ± 0.71	0.1961 ± 0.0635	0.5391 ± 0.1195

While all methods showed slightly degraded performance under optical non-idealities compared to the ideal condition, GRAPHYCS exhibited the least degradation in aberration estimation and object estimation. Our graph-based formulation, when combined with self-calibration, demonstrates its effectiveness in reducing mismatches between the computational model and the physical optical system, enabling more accurate and robust wavefront aberration estimation and correction. The relationship between system misalignment magnitude and wavefront estimation accuracy was further characterized through additional simulations, which showed that GRAPHYCS maintained lower errors while GRAPHYCS without self-calibration exhibited higher wavefront estimation errors as the misalignment increased (Figure S4). In simulations with different underlying aberrations under the same non-ideal conditions, GRAPHYCS consistently outperformed GRAPHYCS without self-calibration, with the performance difference depending on the underlying wavefront aberrations, further supporting the effectiveness of self-calibration (Figure S7).

### GRAPHYCS enables accurate correction of system- and sample-induced aberrations

To validate the capability of GRAPHYCS to estimate and correct optical aberrations, we experimentally evaluated its performance using a wide-field AO setup. We implemented a custom wide-field fluorescence microscope equipped with a DM placed conjugate to the objective’s pupil plane (Methods). This configuration allowed us to both introduce additional known aberrations and apply corrective wavefront.

**Figure 3:**
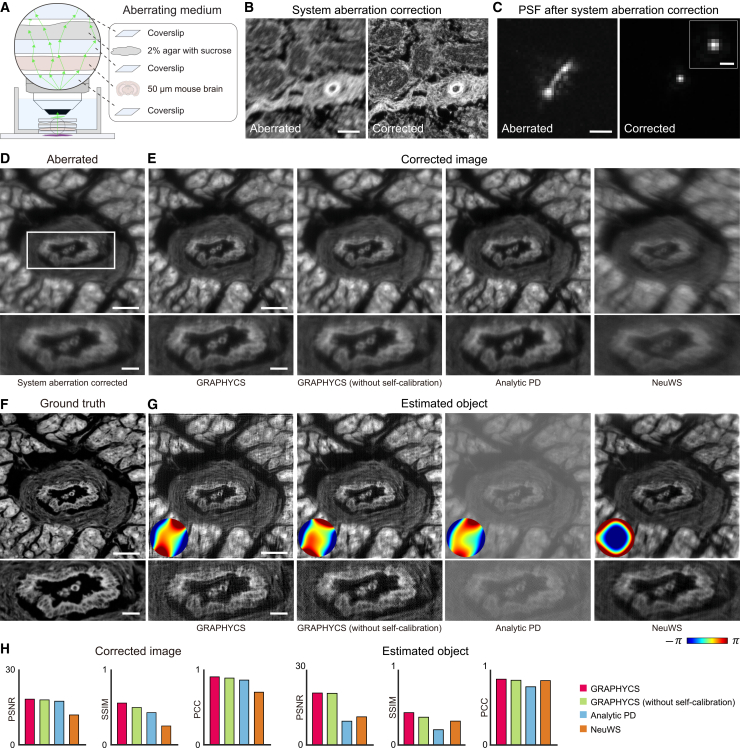
Correction of system- and sample-induced aberrations (A) Schematic of the experimental setup for introducing sample-induced aberrations. The aberrating medium consisted of a 50 μm thick mouse brain slice, 2% agar containing 50% w/v sucrose, and coverslips, placed in a glass-bottom Petri dish with the sample slide positioned below. (B) System aberration correction using GRAPHYCS. Aberrated image of lymph node sample before correction (left) and aberration-corrected image (right). Scale bars, 50 μm. (C) Point spread function (PSF) measurements using a 0.5 μm-sized fluorescent bead before (left) and after (right) system aberration correction. Scale bars, 5 μm. Magnified inset of the corrected PSF. Scale bars, 2 μm. (D) Sample-induced aberration in pancreas tissue. Aberrated image acquired after system aberration correction and prior to sample-induced aberration correction. Scale bars, 50 μm. A magnified view of the boxed region is presented below. Scale bars, 20 μm. (E) Aberration-corrected images of GRAPHYCS, GRAPHYCS without self-calibration, analytic PD, and NeuWS (from left to right), showing correction of the sample-induced aberration shown in (D). Scale bars, 50 μm. A magnified view of the boxed region in (D) is presented below. Scale bars, 20 μm. (F) Ground truth image acquired with a spinning disk confocal microscope. Scale bars, 50 μm. A magnified view of the boxed region in (D) is presented below. Scale bars, 20 μm. (G) Estimated object images of GRAPHYCS, GRAPHYCS without self-calibration, analytic PD, and NeuWS (from left to right) along with their corresponding estimated wavefront aberrations. Scale bars, 50 μm. A magnified view of the boxed region in (D) is presented below. Scale bars, 20 μm. (H) Quantitative comparison of image quality metrics (PSNR, SSIM, and PCC) for the aberration-corrected images (left) and the estimated object images (right).

For our imaging experiments, we used commercial microscope slides of lymph node and pancreas tissue. To introduce sample-induced aberrations, we prepared an aberrating medium consisting of a 50 μm thick mouse brain slice, 2% agar containing 50% w/v sucrose, and coverslips (Figure 3A). This aberrating medium was placed in a glass-bottom Petri dish, with the sample slide positioned below the medium. This arrangement created phase distortions by introducing multiple layers with heterogeneous refractive indices (Figure S8).

We first verified the ability of GRAPHYCS to compensate system aberrations inherent to our optical setup, such as those arising from the non-flat surface of the DM.^37^ While GRAPHYCS can simultaneously estimate both system- and sample-induced aberrations within a unified optimization, we performed a separate correction of the static system aberration to isolate the effects of sample-induced aberrations. We estimated the system aberrations using the set of diversity images acquired from the lymph node sample with a field of view of 274 μm × 274 μm (512 × 512 pixels), and applied the corresponding corrective wavefront via the DM (Figure 3B; Figure S9). The measured iPSF after correction showed significant improvement compared to the uncorrected iPSF (Figure 3C). The full-width at half-maximum (FWHM) values of the corrected iPSF along the *x* and *y* axes were approximately 1.07 μm and 1.07 μm, respectively.

After correcting system aberrations, we next evaluated the performance of GRAPHYCS in compensating sample-induced aberrations. Using the pancreas tissue sample slide placed beneath the aberrating medium, we acquired the aberrated image and diversity images with the same field of view (274 μm × 274 μm). The aberrated image of the pancreas sample showed degraded resolution and contrast due to sample-induced aberrations (Figure 3D; Figure S8). To assess the improvement in aberration corrections, we compared the four methods used in the simulation experiments on the acquired datasets: (1) GRAPHYCS, (2) GRAPHYCS without self-calibration, (3) analytic PD, and (4) NeuWS. After characterizing the sample-induced wavefront distortion, we applied the corresponding corrective wavefront estimated by each method to the DM. GRAPHYCS demonstrated improved performance for aberration correction compared to the other methods. We observed that GRAPHYCS achieved enhanced resolution and contrast, clearly resolving structural details that remained blurred in the aberration-corrected images from GRAPHYCS without self-calibration, analytic PD, and NeuWS (Figure 3E).

To evaluate the effectiveness of GRAPHYCS in object estimation, we conducted a comparative analysis using the same dataset. To obtain a ground truth image, we acquired the image of the same pancreas tissue sample slide after removal of the aberrating medium using a commercial spinning disk confocal microscope (Figure 3F). Visual assessment of the estimated object revealed improvements with GRAPHYCS compared to the other methods (Figure 3G). It restored fine structural features in the estimated object images, which were degraded in the results from GRAPHYCS without self-calibration, analytic PD, and NeuWS. The estimated objects appeared visually sharper than the corrected images because they corresponded to the deconvolution outcome.

We quantitatively evaluated aberration correction performance using PSNR, SSIM, and PCC by comparing the aberration-corrected images to the spinning disk confocal microscope image as a ground truth image. In addition, we assessed the object estimation performance using the same metrics (PSNR, SSIM, and PCC) by comparing the estimated object images to the same spinning disk confocal microscope image. GRAPHYCS led to improved performance compared to GRAPHYCS without calibration, analytic PD, and NeuWS across all metrics (Figure 3H). For the aberration-corrected images, based on the quantitative evaluation, GRAPHYCS consistently outperformed the other methods across all metrics. It achieved a PSNR of 18.33 dB, SSIM of 0.5600, and PCC of 0.9079, compared to GRAPHYCS without self-calibration, analytic PD, and NeuWS (Table 4). Similarly, for the estimated object images, GRAPHYCS demonstrated higher metric values, compared to GRAPHYCS without self-calibration, analytic PD, and NeuWS (Table 4). We note that these quantitative metrics are constrained by differences in imaging modalities, specifically confocal microscopy for the ground truth and wide-field microscopy for the evaluated images. To further validate the optical correction performance, we additionally evaluated wide-field images acquired after removal of the aberration medium (Figure S10) and performed an additional experiment using a gelatin and sucrose medium placed between coverslips to introduce refractive-index mismatches with the surrounding water (Figure S11). This additional experiment further supported the effectiveness of GRAPHYCS in correcting sample-induced aberrations.

**Table 4:** Quantitative comparison of aberration correction and object estimation in wide-field microscopy

Method	Aberration-corrected image			Estimated object		
	PSNR (dB)	SSIM	PCC	PSNR (dB)	SSIM	PCC
GRAPHYCS	18.33	0.5600	0.9079	20.91	0.4344	0.8825
GRAPHYCS (without self-calibration)	18.05	0.5018	0.8919	20.79	0.3730	0.8678
Analytic PD	17.52	0.4339	0.8651	9.61	0.2070	0.7739
NeuWS	12.08	0.2578	0.7057	11.34	0.3208	0.8635

### Spatially varying aberration sensing across the large field of view

Existing computational wavefront sensing methods, including analytic PD^38^ and NeuWS,^39^ rely on the assumption that aberrations are spatially invariant within the imaging area. While this assumption holds in small or localized regions, it becomes a limiting factor when imaging biological samples that exhibit spatially varying refractive index distributions across the entire field of view. To address this challenge, we implemented GRAPHYCS to estimate spatially varying aberrations over large imaging areas, as aforementioned and illustrated in Figure 1D.

To experimentally validate the capability of our spatially variant model, we used the same pancreas sample and aberrating medium described earlier, but expanded the field of view to 1094 μm × 1094 μm (2048 × 2048 pixels). This larger imaging area contained spatially varying distortions due to the heterogeneous nature of the aberrating medium, leading to non-uniform degradation in image quality across the field of view. We conducted a comparative analysis between two approaches: (1) GRAPHYCS with a spatially variant model, and (2) GRAPHYCS with a spatially invariant model, which estimates a single set of aberration coefficients for the entire field of view. We excluded analytic PD and NeuWS from the comparison because they were not able to process the image due to a memory issue (Table S3).

**Figure 4:**
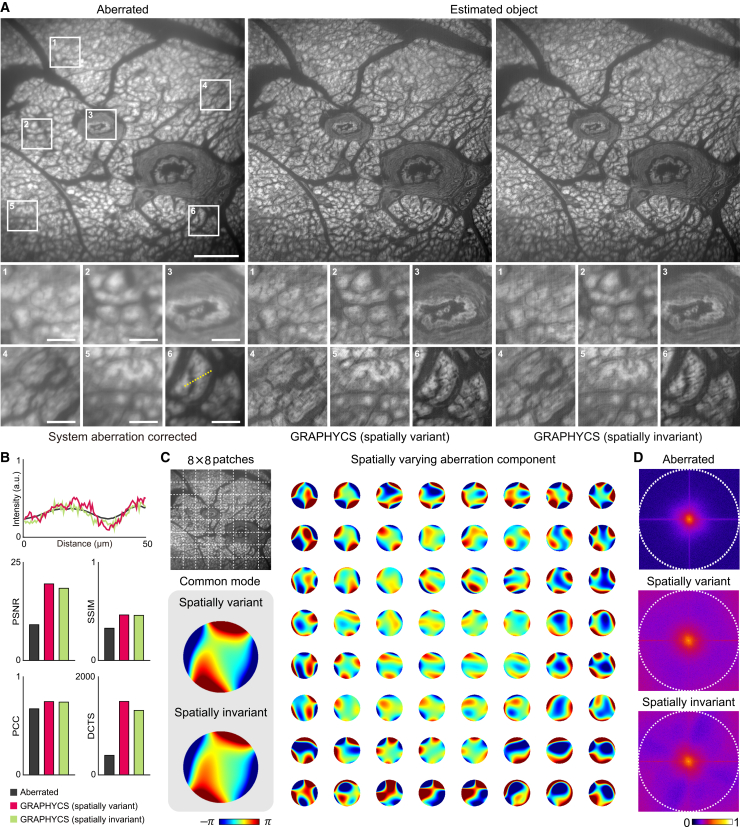
Spatially varying aberration sensing across the large field of view (A) Object estimation results from spatially variant and spatially invariant models. Aberrated image of pancreas tissue acquired through the aberrating medium after system aberration correction and prior to sample-induced aberration correction with a field of view of 1094 μm × 1094 μm (left), and estimated object images using GRAPHYCS with the spatially variant model (center) and the spatially invariant model (right). Scale bars, 200 μm. Magnified views of the boxed regions are presented below. Scale bars, 50 μm. (B) Line intensity profiles extracted along the yellow dashed line within the magnified region in (A), demonstrating improved contrast in the estimation from the spatially variant model (top). Comparison of image quality metrics (PSNR, SSIM, PCC and DCTS) for the estimated objects across the entire field of view, showing PSNR and SSIM (middle), PCC and DCTS (bottom). (C) Visualization of spatially variant aberration estimation. The entire field of view was divided into 8 × 8 patches for spatially variant aberration estimation (left top). Estimated common mode aberration components affecting the entire field of view from the spatially variant model (left middle) and the spatially invariant model (left bottom). Estimation of the spatially varying aberration component from different patches showing local variations in wavefront distortion (right). (D) Frequency domain analysis of object estimation. Fourier spectra of the aberrated image (top), estimated object images using the spatially variant model (middle), and the spatially invariant model (bottom). The spatial frequency content of the estimated object using the spatially variant model demonstrated enhanced high-frequency content compared to both the aberrated image and the spatially invariant model result, indicating improved recovery of fine structural details across the large field of view. The dashed circles indicate the diffraction-limit frequency (1.169 μm^−1^).

For qualitative evaluation, we compared the estimated objects obtained from the spatially variant and spatially invariant models across multiple regions to the image of the same pancreas tissue sample slide obtained by a commercial spinning disk confocal microscope as a ground truth image. Structural comparison was performed using only confocal images due to the difficulties in repositioning the sample to match the identical large fields of view of the wide-field microscopy images after removal of the aberrating medium. Visual inspection showed that the spatially variant model achieved improved object recoveries, with fine structural features more clearly resolved across the entire imaging field of view. Magnified insets of selected regions further highlighted these improvements (Figure 4A). Line intensity profiles extracted from the first region demonstrated that the estimated object using GRAPHYCS with a spatially variant model showed improved contrast compared to both the spatially invariant model and the aberrated image (Figure 4B). To quantify this improvement, we evaluated the set of image quality metrics (PSNR, SSIM, and PCC) for the estimated objects across the entire field of view. Additionally, we evaluated a normalized discrete cosine transform Shannon entropy (DCTS)^42^ across the entire field of view. GRAPHYCS with the spatially variant model achieved higher PSNR, SSIM, and PCC scores compared to the spatially invariant model and aberrated image (Table 5). Similarly, it exhibited higher values in the DCTS metrics compared to the spatially invariant model and aberrated image. The results from the spatially variant model demonstrated clear enhancements compared to both the aberrated image and the spatially invariant model (Figure 4B).

**Table 5:** Quantitative comparison between spatially invariant and spatially variant aberration estimation

Method	PSNR (dB)	SSIM	PCC	DCTS
Aberrated image	9.17	0.3303	0.6737	403.8
GRAPHYCS (spatially invariant)	18.40	0.4604	0.7416	1315.4
GRAPHYCS (spatially variant)	19.51	0.4645	0.7467	1497.0

The estimated wavefront map visualized the spatial variation of aberrations across the field of view. The common mode component identified general trends affecting the entire field, while the spatially varying components captured local phase deviations (Figure 4C). We note that these localized aberrations cannot be effectively corrected by a single, global estimate. In addition, we performed a frequency domain analysis on the estimated object images over the entire field of view. The spatial frequency content of the spatially variant model result demonstrated the enhanced high-frequency content compared to both the aberrated image and the spatially invariant estimation, indicating improved recovery of fine structural details (Figure 4D). We further compared GRAPHYCS with the spatially variant model and patch-wise analytic PD (Figure S12). The patch-wise analytic PD approach resulted in less accurate estimations of both the aberration and the object and boundary artifacts. In contrast, GRAPHYCS with the spatially variant model showed more accurate phase maps and clearer structural recovery by leveraging information from the entire field of view through joint optimization of common mode and spatially varying components, as well as the overlap-save method used in the spatially varying image formation model.

### Aberration correction in light-sheet microscopy of dynamic samples with GRAPHYCS

**Figure 5:**
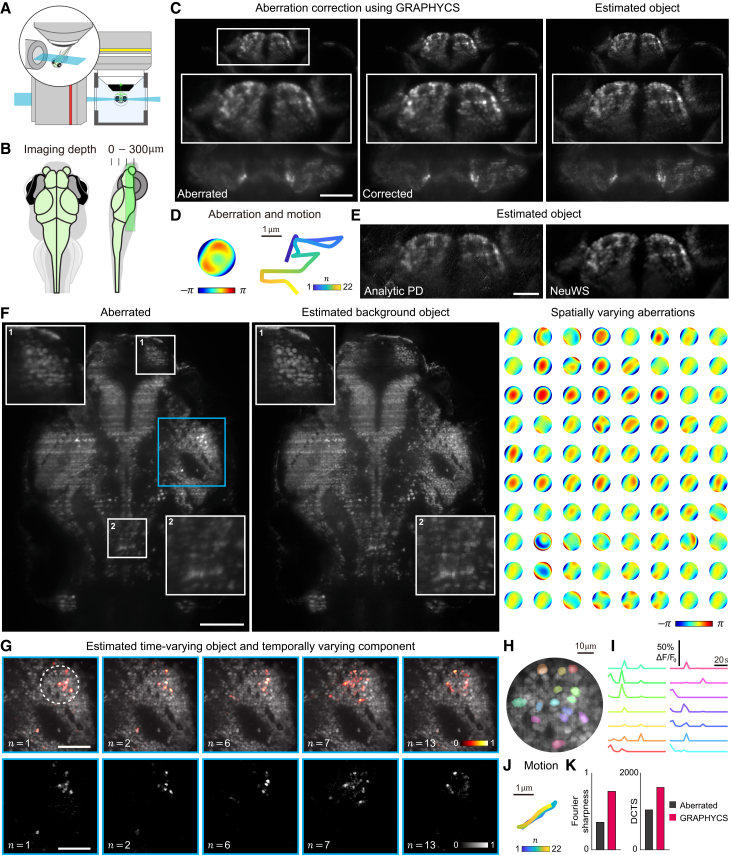
Aberration correction in light-sheet microscopy of the living larval zebrafish brain with GRAPHYCS (A) Schematic of the light-sheet imaging setup. (B) Schematic of larval zebrafish brain showing the imaging depth range (200 μm–300 μm). (C) Aberration correction using GRAPHYCS in larval zebrafish (6 dpf) brain expressing pan-neuronal nuclear localized GCaMP6s at 300 μm depth. Aberrated image acquired prior to sample-induced aberration correction (left), aberration-corrected image (center), and object estimation (right). Scale bars, 50 μm. (D) Estimated wavefront aberration (left) and motion trajectories (right), showing frame-wise motion trajectories with colors encoding the phase-diversity acquisition sequence (*n*). (E) Object estimation results from analytic PD (left) and NeuWS (right). Scale bars, 20 μm. (F) Spatially varying aberration estimation in larval zebrafish (4 dpf) brain expressing pan-neuronal nuclear-localized GCaMP6s at 200 μm depth. Aberrated image acquired prior to sample-induced aberration correction (left), estimated background object (center), and visualization of estimated spatially variant aberrations (right). The field of view was divided into 10 × 8 patches. Scale bars, 100 μm. (G) Estimation of time-varying object and temporally varying component. Magnified views corresponding to the blue boxed region in (F). Time-varying object images (top) showing neuronal activity overlaid on the estimated background object at multiple time points in the phase-diversity sequence (*n*), with corresponding temporally varying components (bottom) estimated by GRAPHYCS. Scale bars, 50 μm. (H) Temporal maximum intensity projection (MIP) of the estimated time-varying object with randomly selected neurons overlaid as regions of interest. Magnified views corresponding to the dotted circular region in (G). (I) Extracted neuronal activity traces for the neurons indicated in (H) with the color of each trace matched to the corresponding neuron. (J) Estimated motion from a spatially variant model, showing frame-wise motion trajectories with colors encoding the phase-diversity acquisition sequence (*n*). (K) Quantitative comparison between aberrated and estimated background object images using image quality metrics (Fourier sharpness and DCTS) across the entire field of view, showing Fourier sharpness (left) and DCTS (right).

To evaluate the effectiveness of GRAPHYCS for aberration correction in dynamic samples, we performed light-sheet microscopy of a larval zebrafish brain *in vivo* with motion and brain activity. The light-sheet microscope was implemented by integrating light-sheet illumination into our wide-field AO setup (Methods). We next experimentally validated the capability of our model for time-varying objects as illustrated in Figure 1E, using the same PD acquisition protocol as in the wide-field imaging experiments. In addition, we employed a triangular driving signal for the acquisition, which was designed to acquire the images with no applied defocus multiple times during the PD acquisition sequence (Methods). For *in vivo* light-sheet imaging experiments, we imaged larval zebrafish (4–6 days post-fertilization) brains expressing pan-neuronal nuclear-localized GCaMP6s at imaging depths of 200 μm–300 μm (Figures 5A and 5B).

We first verified that GRAPHYCS effectively compensates sample-induced aberrations at a depth of 300 μm in the larval zebrafish brain (Figure 5C). The neuronal nuclei in the aberrated image appeared blurred due to the accumulated wavefront distortions from tissue heterogeneities at this imaging depth. We applied GRAPHYCS to the acquired image set to jointly estimate the aberrations and the underlying object structure. The optimization converged in 500 epochs, resulting in the aberration coefficients that characterized the sample-induced wavefront distortion and trajectories of specimen motion, which were on a scale comparable to the cell body size, during the image acquisition (Figure 5D). We observed enhanced resolution and contrast achieved by applying the corresponding corrective wavefront estimated by GRAPHYCS to the DM. Individual neuronal nuclei were clearly resolved with well-defined boundaries in the corrected image, demonstrating effective aberration correction in the live larval zebrafish brain (Figure 5C). Visual assessment of the estimated object revealed improvements, with the restoration of fine structural features (Figure 5C). We compared the estimated object with analytic PD and NeuWS on the corresponding cropped region from the same dataset. GRAPHYCS recovered nuclear boundaries with higher local contrast, while the analytic PD and NeuWS appeared comparatively blurred (Figure 5E).

In addition, we imaged the sample at a depth of 200 μm where neurons are distributed over a larger field of view that was divided into 10 × 8 patches. The optimization converged in 500 epochs, resulting in the spatially varying aberration coefficients, background object, and temporally varying components that characterized both the sample-induced wavefront distortion and time-varying changes in neuronal activity. The estimated background object showed improvement over the aberrated image, with individual neuronal nuclei clearly resolved and enhanced contrast with the restoration of fine structural features (Figure 5F). The estimated spatially varying aberrations exhibited spatial variability across the zebrafish brain, reflecting tissue refractive-index inhomogeneity (Figure 5F). The estimated time-varying object captured neuronal activity traces with single-neuron resolution (Figure 5G and Video S1). To visualize these traces, we obtained a temporal maximum intensity projection of the estimated time-varying object, manually segmented neuronal regions of interest, and extracted activity traces for randomly selected neurons (Figures 5H and 5I). The extracted signals showed neuron-specific temporal activity patterns, indicating that neuronal activity patterns are preserved in the time-varying object estimated by GRAPHYCS. Our model also estimated frame-wise motion parameters that captured specimen drift, and the resulting trajectories varied across samples, reflecting sample-to-sample differences in motion (Figures 5D and 5J). For quantitative assessment, we evaluated DCTS and a Fourier transform-based sharpness metric^27^ for the estimated objects across the entire field of view (Figure 5K). GRAPHYCS achieved higher values in the DCTS (DCTS of 1619.5) and the Fourier sharpness metrics (Fourier sharpness of 0.7578) compared to the aberrated image (DCTS: 1035.9, Fourier sharpness: 0.3564). These results demonstrate that our approach enables simultaneous spatially varying aberrated wavefront sensing and time-varying object estimation, effectively addressing the limitations of wavefront sensing methods that assume a static object over all acquired images, while the current total acquisition time of approximately 90 s limits the ability to capture fast dynamic phenomena.

Video S1. Estimation of time-varying object in living zebrafish brain using GRAPHYCS

## Discussion

In this study, we introduced GRAPHYCS, a computational AO framework that formulates optical imaging as a differentiable computational graph to overcome three fundamental limitations of existing methods: performance degradation under system non-idealities, restriction to spatially invariant aberrations, and inability to handle dynamic samples.

Self-calibration in our wavefront sensing framework addresses the critical mismatch between idealized computational models and real optical systems. Our results demonstrate that self-calibration can improve aberration estimation accuracy, with up to a 9-fold improvement in wavefront RMS error under system imperfections compared to analytic PD. Under near-ideal conditions, the aberration can be accurately estimated by the model without self-calibration, leading to comparable correction results; however, as system misalignment increases, GRAPHYCS without self-calibration shows a progressive increase in wavefront estimation error (Figure S4). This capability eliminates the need for separate calibration procedures and proves particularly valuable where perfect system alignment cannot be guaranteed.

Spatial variation through patch-wise modeling extends computational AO beyond traditional assumptions of uniform aberrations. While analytic PD can be applied to spatially segmented regions, with separate optimizations performed for each patch and the results stitched together to form a full field of view image, each segmented region is optimized independently under a locally invariant assumption, and no explicit constraint enforces global consistency across neighboring patches during optimization. As a result, boundary artifacts may arise when aberrations vary continuously across the field of view, leading to less accurate and spatially inconsistent wavefront estimations across adjacent regions (Figure S12). Furthermore, patch-wise analytic PD is fundamentally constrained by the limited amount of information accessible to each localized optimization, as parameters are derived from a smaller field of view, resulting in reduced data density and often leading to less accurate estimations of both the wavefront aberration and the underlying object structure. In contrast, GRAPHYCS leverages information from the entire field of view to estimate the primary common-mode aberrations and the overlap-save implementation used in the image formation model. By utilizing the full informational content of the dataset for the majority of the phase distortion, our framework enables a more robust and accurate estimation and alleviates the boundary artifact. Our experimental validation across fields of view exceeding 1 mm^2^ shows improved object estimation quality in samples with heterogeneous refractive index distributions. This addresses a common limitation in biological imaging where aberrations vary significantly across imaging areas.

Temporal dynamics through object decomposition enables aberration correction in live biological specimens—a paradigm shift for computational AO. Our demonstration in live larval zebrafish brain imaging shows successful simultaneous aberration estimation and dynamic activity detection, opening applications previously limited to sensor-based approaches with complex hardware requirements.

These capabilities collectively address the major barriers limiting widespread adoption of computational AO. By handling system imperfections, spatial heterogeneity, and temporal dynamics, GRAPHYCS can be implemented on existing microscope platforms by simply adding a DM. This converts virtually any fluorescence microscope into an AO system capable of robust aberration correction without requiring precise system calibration, uniform sample properties, or sample immobilization.

We note that the underlying physics and core image formation models are shared between GRAPHYCS and analytic PD, as both rely on established Fourier optics principles to describe incoherent imaging. Since the underlying object and aberration parameters are shared across all diversity images, the optimization is constrained by the multiple input-output pairs rather than a single observation, reducing the ambiguity between object structure and aberrations. However, analytic PD is fundamentally constrained by its reliance on a fixed image formation model coupled with fixed, closed-form equations for numerical optimization. While it may be possible to accommodate minor variations within such a framework, any meaningful change to the image formation model—such as the inclusion of system misalignments, self-calibration, or sample motion—necessitates a complete manual re-derivation of all mathematical expressions and gradients. Furthermore, analytic PD requires the explicit computation and inversion of a pseudo-Hessian matrix, which is mathematically cumbersome and limits the optimization’s scalability as parameter complexity increases. In contrast, GRAPHYCS represents the image formation model as a chain of modular and differentiable components within a unified computational graph. This architecture enables automatic differentiation through backpropagation, similar to modern neural network frameworks, allowing the system to compute gradients efficiently across all nodes. This modularity makes it extremely easy to reflect system-specific non-idealities or complex dynamics directly into the image formation model, enabling the joint optimization of all parameters. Consequently, the extendibility of GRAPHYCS is superior to previous methods, as new physical effects can be incorporated without reformulating the underlying engine. Crucially, our approach eliminates the need for explicit Hessian calculation, making the joint optimization of high-dimensional non-idealities computationally far more tractable and efficient for real-world biological imaging. As supported by the results in Table S3, GRAPHYCS provides greater robustness under experimental conditions, leading to more stable and often faster convergence, whereas analytic PD can converge faster in idealized simulation settings. The aberration estimation performance and computational cost differences across dataset configurations are summarized in Figure S13 (image count), S14 (PD strategies), and Table S4 (image size). We note that the choice of PD configuration strongly affects the performance of NeuWS, which requires at least 100 images with random phase modulations for reliable wavefront estimation,^43^^,^^44^ whereas GRAPHYCS achieves accurate estimation under the defocus-based diversity protocol used in this study (Figure S3; Table S1).

Several opportunities remain for further development. Expanding beyond the current Zernike polynomial basis could enhance the correction of high-order aberrations in complex biological samples. Extending to three-dimensional volumetric imaging would enable aberration correction throughout sample depth, critical for thick specimens with depth-varying aberrations. Integration with optical sectioning techniques such as confocal or multiphoton microscopy, which can provide a scanning-based optical correction scheme, could broaden applicability while preserving computational AO advantages.

### Limitations of the study

GRAPHYCS offers a computational graph-based perspective on the expression of image formation and opens future directions for computational imaging in complex environments. While we have demonstrated that modeling the image formation as a differentiable computational graph simultaneously enables self-calibration, spatially variant wavefront sensing across a large field of view, and aberration correction with object estimation for dynamic biological samples, it is important to recognize its limitations. The proposed framework relies on a two-dimensional image formation model, in which the imaging sample is treated as a thin object and out-of-focus contributions from three-dimensional structures are not explicitly modeled. This assumption can be violated in thick three-dimensional biological samples and wide-field imaging conditions with high levels of out-of-focus background, where three-dimensional PD modeling would be more appropriate. Under such conditions, the out-of-focus background from three-dimensional structures can introduce discrepancies between the reconstructed object and the underlying object structure, which may contribute to the performance gap relative to the ground truth observed in Figure 3. Extending the current framework to incorporate a fully three-dimensional image formation model and PD formulation will be an important direction for future work.

In this work, we used a single Zernike mode (Z5) to introduce phase diversity. The number of Zernike modes used for aberration correction was limited to 12 due to the hardware constraints of the DM. While these design choices were sufficient to demonstrate the capabilities of GRAPHYCS, exploring alternative or combined diversity modes and incorporating higher-order aberration modes may further improve the sensitivity and robustness of aberrated wavefront sensing across diverse imaging conditions. In principle, increasing the number of Zernike modes would allow representation of higher-order aberrations and may provide additional correction capability. However, incorporating higher-order modes increases the number of learnable parameters (i.e., additional Zernike coefficients to be optimized), which enlarges the parameter space and can reduce optimization stability. Reliable estimation of a larger number of modes would also require a greater number of PD images to ensure stable and well-conditioned parameter estimation.

The current implementation also presents a practical limitation in temporal resolution rather than a fundamental constraint of the GRAPHYCS algorithm itself. The total PD acquisition time of approximately 90 s and the ∼3.2-s inter-frame interval, arising from the exposure time per frame, camera readout, DM control, and software overhead in our hardware setup, limit the ability to capture fast dynamic phenomena such as rapidly changing neural activity. Advances in imaging techniques and acquisition strategies, such as incorporating brighter fluorescent calcium indicators, high-sensitivity sCMOS cameras, faster acquisition and control pipelines, and optimized PD strategies requiring fewer diversity images, could reduce the total acquisition time.

## Resource availability

### Lead contact

Requests for further information and resources should be directed to and will be fulfilled by the lead contact, Dr. Young-Gyu Yoon (ygyoon@kaist.ac.kr).

### Materials availability

The zebrafish line used in this study is available from the lead contact upon reasonable request.

### Data and code availability

• The datasets generated and/or analyzed during the current study have been deposited at Zenodo at https://zenodo.org/records/17049780 and are publicly available as of the date of publication.
• All original code has been deposited at a GitHub repository and is publicly available at https://github.com/NICALab/GRAPHYCS as of the date of publication.
• Any additional information required to reanalyze the data reported in this paper is available from the lead contact upon request.

## Acknowledgments

This research was supported by the 10.13039/501100003725National Research Foundation of Korea (NRF) (RS-2026-25475246, RS-2026-25504129, RS-2023-00209473, RS-2023-00264409, RS-2023-00264980, RS-2022-NR068424), the 10.13039/501100003716Korea Basic Science Institute (National Research Facilities and Equipment Center) grant funded by the Ministry of Science and ICT (RS-2024-00401676), Bio and Medical Technology Development Program through the NRF funded by the Ministry of Science and ICT (RS-2021-NR056586), the BK21 plus program through the NRF funded by the Ministry of Education of Korea, a Research grant from HFSP (RGP003/2024) with the award DOI (https://doi.org/10.52044/HFSP.RGP0032024.pc.gr.194148), the 10.13039/501100007107KAIST Quantum+X Convergence R&D Project, and the KAIST C2(Creative and Challenging) Project.

## Author contributions

E.-S.C. and J.P. designed the computational adaptive optics algorithm with contributions from Y.C. and M.E. E.-S.C., and J.P. performed experiments and analyzed data. E.-S.C. designed and implemented wide-field and light-sheet adaptive optics microscope systems with contributions from M.E. H.J. performed adaptive optics experiments with multiphoton excitation under the supervision of J.-H.P. H.S. prepared mouse brain slice samples under the supervision of J.-B.C. E.-S.C., J.P., and Y.-G.Y. wrote the manuscript with input from all authors. Y.-G.Y. conceived and led this work.

## Declaration of interests

Y.-G.Y. and J.-B.C. are co-founders, shareholders, and employers of a company specializing in various imaging services; this research was conducted independently and is not affiliated with the company.

## STAR★Methods

### Key resources table

**Table:** 

REAGENT or RESOURCE	SOURCE	IDENTIFIER
**Biological samples**		

Lymph node microscope slide	DIY-SCIENCE	Commercial microscope slide
Pancreas tissue microscope slide	USCAMEL	Commercial microscope slide
Mouse brain slices (fixed, 50 μm)	This paper	N/A

**Deposited data**		

GRAPHYCS simulation and imaging data	This paper	https://zenodo.org/records/17049780

**Experimental models: Organisms/strains**		

Tg(HuC:H2B-GCaMP6s)	This paper	N/A

**Software and algorithms**		

GRAPHYCS source code	This paper	https://github.com/NICALab/GRAPHYCS
MATLAB	Mathworks	https://www.mathworks.com/products/matlab.html
Zemax OpticStudio	Ansys	https://www.ansys.com/products/optics/ansys-zemax-opticstudio
Python	Python	https://www.python.org/
PyTorch	Meta AI	https://pytorch.org/
Numpy	Numpy	https://numpy.org/
scikit-image	scikit-image	https://scikit-image.org/
SciPy	SciPy	https://scipy.org/
CUDA	Nvidia Corporation	https://developer.nvidia.com/cuda-toolkit
Analytic PD	Johnson et al.^38^	https://github.com/ceej640/PhaseDiversity
NeuWS	Feng et al.^39^	https://github.com/Intelligent-Sensing/NeuWS
SUPPORT	Eom et al.^45^	https://github.com/NICALab/SUPPORT

### Experimental model and study participant details

#### Mouse brain slice preparation

Mouse brain slices were prepared from wild-type male C57BL/6J mice at 7 weeks of age. Mice were housed in ventilated cages under standardized environmental conditions with 12 h light/dark cycle at 20°C–24 °C and 40–60% humidity. Mice were deeply anesthetized with isoflurane and perfused transcardially with phosphate-buffered saline (PBS) followed by 4% paraformaldehyde (PFA) in PBS. Brains were post-fixed in 4% paraformaldehyde at 4°C for 2 h. Afterward, the brains were sectioned at a thickness of 50 μm using a vibratome (Leica VT1000S). The brain slices were stored in 1×PBS solution until use. These brain slices served as part of the aberrating medium in our experimental design to introduce spatially varying phase distortions. All animal procedures were approved by the Institutional Animal Care and Use Committee (IACUC) of KAIST (approval no. KA2025-063-v1).

#### Zebrafish husbandry

All animal experiments involving zebrafish (Danio rerio) were conducted in accordance with the guidelines for animal research approved by the Institutional Animal Care and Use Committee (IACUC) of KAIST (KA-2021-125). All larvae were maintained on a 10-h dark, 14-h light cycle at 28°C until at least 6 days post fertilization (dpf). The sex of the larvae is not specified at this developmental stage and was therefore not determined.

### Method details

#### Wide-field adaptive optics system description

We implemented a wide-field fluorescence microscope integrated with a piezo deformable mirror (DM) as shown in Figure 1B. For excitation, we used a blue collimated LED light source (λ = 470 nm, M470L3-C2, Thorlabs) which was focused by an achromatic lens (L1, f = 150 mm, AC508-150-A, Thorlabs). The imaging system utilized a 10×, 0.3 NA water dipping objective lens (O1, CFI Plan Fluor 10XW, Nikon) for all experiments. Emitted fluorescence was collected through the same objective, separated from excitation light using a dichroic mirror (F1, MD498, Thorlabs), and filtered through an emission filter (MF525-39, Thorlabs) before detection. The emitted fluorescence light then passed through a 4f relay system consisting of two achromatic lenses: L2 (f = 180 mm, AC508-200-A, Thorlabs) and L3 (f = 150 mm, AC254-150-A, Thorlabs). This configuration provided a 1.2:1 demagnification of the pupil plane, reducing the beam diameter from approximately 12 mm–10 mm to match the active area of the deformable mirror. The deformable mirror (DMP40/M-P01, Thorlabs) with 40 actuators and Ø10.0 mm pupil size was placed at the Fourier plane of this relay system at approximately 7.5-degrees incidence angle. After reflection from the DM, the emitted light was focused through a camera tube lens (L4, TTL200-A, Thorlabs) onto a scientific CMOS camera (Zyla 5.5 sCMOS, Andor, Oxford Instruments). For image acquisition and adaptive optics control, we developed custom image acquisition software in MATLAB that integrated the manufacturers' SDKs to synchronize and operate both the deformable mirror and sCMOS camera. For acquiring phase-diversity images, custom-designed coefficient sequences implemented in our software mitigated hysteresis effects while ensuring consistent imaging conditions (Figure S15). The resulting pixel size in the sample plane was 0.53 μm. The 3D rendering of the optical setup implementation is shown in Figure S16.

#### Light-sheet adaptive optics system description

We implemented a light-sheet adaptive optics system by integrating light-sheet illumination into our wide-field adaptive optics setup. To generate a light-sheet, we used a 488 nm laser (Cobolt 06-MLD, HÜBNER Photonics) and a Powell lens (P1, 43–473, Edmund Optics, fan angle = 30°) for a uniform light-sheet illumination. The diverging beam from the Powell lens was focused into a line after passing through a plano-convex cylindrical lens (C1, f = 50 mm, LJ1695RM-A, Thorlabs). After passing through the second plano-convex cylindrical lens (C2, f = 75 mm, LJ1703RM-A, Thorlabs), the excitation light passed through a 4f relay system consisting of two achromatic lenses (R1, f = 60 mm, AC254-60-A, Thorlabs and R2, f = 150 mm, AC254-150-A, Thorlabs) to the back pupil plane of an illumination objective (O2, f = 45 mm, AmScope Plan Achromat 4×, 0.10 NA). The light-sheet generated by the illumination objective (O2) illuminated the specimen orthogonally to the detection objective (O1). For sample mounting, we used a custom 3D printed sample chamber and specimen holder (Figure S17).

#### Synthetic data generation

To evaluate the performance of GRAPHYCS, we first generated synthetic wide-field microscopy data using commercial optical simulation software (Zemax OpticStudio) and then added Poisson and Gaussian noise to the resulting images. In the optical simulation software, we designed an optical model of our wide-field microscope with the same specifications as our experimental setup (Figure S1). To simulate aberrated images, a Zernike surface was placed at the position of the DM, conjugate to the objective lens pupil plane, and wavefront aberrations were applied using randomly generated Zernike coefficients. The 12 Zernike modes from primary astigmatism to tetrafoil (excluding piston, tip, and tilt) controllable by the DM hardware were used. The coefficient values were randomly sampled from a uniform random distribution in the range ±0.4 μm to create phase distortions. Synthetic datasets were created in the absence and presence of real-world system imperfections using this model. We deliberately introduced system non-idealities by shifting the DM surface by either −2.0 mm in the x direction and +1.0 mm in the y direction or −2.5 mm in the x direction and +1.5 mm in the y direction, and tilting the DM surface by 1.0-degrees to replicate misalignment. For phase-diversity acquisitions, defocus (*Z*_5_) was used as the applied phase at the deformable mirror plane, generating an initial aberrated image and 11 diversity images by varying the *Z*_5_ Zernike coefficient with values ranging from −0.44 μm to +0.44 μm in equal steps. We determined the number of phase-diversity images by evaluating the trade-off between aberration estimation accuracy and the number of images (Figure S13) and selected the defocus as diversity phase because defocus-based phase modulation provides consistently stable and accurate wavefront estimation across different phase modulation strategies (Figure S14). The underlying ground truth object used for simulation input was a confocal microscope image of *Penicillium* or larval zebrafish brain, and the raw image was denoised using the SUPPORT^45^ algorithm. The ground truth for the aberration-corrected image was generated using the same optical model under ideal, aberration-free conditions, in which the confocal image was used as the source image. Additional simulation experiments presented in the Supplementary Materials were generated using the forward model implemented in Python based on Fourier optics principles, with a confocal microscope image of a lymph node sample as the ground truth object and a zebrafish calcium imaging dataset^46^ as the temporally varying object for dynamic sample simulations.

#### Spatially variant convolution

To account for field-dependent aberrations while avoiding boundary artifacts, we applied the overlap-save method to combine the spatially varying convolution outputs (Figure S18). Let *w*_*patch*_, *h*_*patch*_ be the patch width and height, where $w_{patch},{\;h}_{patch}=\frac{w}{G_{1}},\frac{h}{G_{2}}$. The image to be convolved was zero-padded by the (*k*-1) and each patch was selected to have a size of (*w*_*patch*_+*k*-1)×(*h*_*patch*_+*k*-1), where neighboring patches overlapped by a length of *k*-1. Our PSF had size (*k*,*k*), *k* = 101 pixels. This can be expressed as multiplying the image with distinct window functions *w*_*i*_(*x*,*y*) to obtain the *i*-th patch having index (*m*_1_,*m*_2_) within the *G*_1_×*G*_2_ patches:

$$w_{i}\left(x,y\right)=\left\{ \begin{array}{cc} 1, & if\;m_{1}w_{patch}<x<{\left(m_{1}+1\right)w}_{patch}+k-1,\\ & \text{and}\;m_{2}h_{patch}<y<{\left(m_{2}+1\right)h}_{patch}+k-1\\ 0, & \text{otherwise} \end{array}\right.\text{.}$$

Spatially varying iPSFs were generated as

$$h_{i}\left(x,y\right)=|F\left\{P\left(u,v\right)e^{j\Phi_{S,i}\left(u,v\right)}\right\}{|}_{f_{u}=\frac{x}{\lambda l},f_{v}=\frac{y}{\lambda l}}^{2}\text{.}$$

Φ_*S*,*i*_(*u*,*v*) is the phase estimation for the *i*-th patch. The convolution *q*_*i*_(*x*,*y*) = *h*_*i*_(*x*,*y*)∗(*o*(*x*,*y*)·*w*_*i*_(*x*,*y*)) was applied to maintain the same size as the patch input (*w*_*patch*_, *h*_*patch*_), which effectively discarded the overlapping regions of length *k*-1 between neighboring *q*_*i*_(*x*,*y*) outputs. The final output was combined as:

$$q\left(x,y\right)=\sum_{m_{1}=0}^{G_{1}-1}\sum_{m_{2}=0}^{G_{2}-1}q_{i}\left(x-m_{1}w_{patch},y-m_{2}h_{patch}\right)\text{.}$$

#### System aberration correction

Prior to conducting imaging experiments, system aberration correction was performed to address inherent aberrations in the optical setup, particularly those arising from the non-flat surface of the DM. This step was essential to isolate the effects of sample-induced aberrations in subsequent experiments. System aberrations were characterized using a set of phase-diversity images of a lymph node sample with a field of view of 274 μm × 274 μm. For phase-diversity acquisition, we applied defocus as the additional known aberration using the deformable mirror. A total of 21 diversity images were acquired with defocus values ranging from −0.65 μm to +0.65 μm in equal steps (for a total of 22 images, including the initial aberrated image), providing sufficient information for robust aberration estimation. The system aberration estimation procedure involved optimizing the object, aberration, and calibration parameters by minimizing the defined loss function between predicted and observed images. To compensate for the system aberrations, we applied a corrective wavefront to the deformable mirror that was the opposite of the estimated aberration (Figure S9). We verified the effectiveness of system aberration correction by measuring the PSF using 0.5 μm fluorescent beads.

#### Sample-induced aberration correction in wide-field microscopy

To evaluate the capability of GRAPHYCS in correcting sample-induced aberrations, we conducted experiments with an aberrating medium placed over a commercial microscope slide of pancreas tissue sample. To introduce additional known aberrations, the deformable mirror was controlled to modulate the wavefront using the defocus (Z_5_) mode. A total of 21 diversity images were acquired by varying the Z_5_ coefficient from −0.65 μm to +0.65 μm in equal steps, resulting in 22 images including the initial aberrated image. This defocus range and number of diversity images were empirically determined to be effective for accurate aberration estimation and object estimation. All phase-diversity images were acquired with an exposure time of 1.00 s to ensure sufficient signal-to-noise ratio, particularly when imaging through the highly aberrating medium. The aberration estimation process began by initializing the object estimate with the aberrated image and setting all Zernike coefficients to zero. The optimization was performed by minimizing our defined loss function, which combines image-domain and Fourier domain errors between the predicted and observed diversity images. For aberration coefficient parameterization, we used 12 Zernike modes from primary astigmatism to tetrafoil (modes 4–15, excluding piston, tip, and tilt). The weight parameters for the loss function components were determined through empirical testing to balance structural fidelity and frequency content recovery. After estimating the aberration coefficients, we applied the corresponding corrective wavefront to the deformable mirror. This corrective wavefront was generated by inverting the phase of the estimated aberration (applying the negative of each Zernike coefficient). For comparative analysis, we utilized four approaches: (1) GRAPHYCS, (2) GRAPHYCS without self-calibration, (3) analytic phase-diversity (PD) method, and (4) NeuWS. For GRAPHYCS without self-calibration, we disabled the learnable calibration parameters while maintaining the same Zernike mode parameterization, allowing us to isolate the impact of the self-calibration component. All methods processed identical phase-diversity datasets for performance evaluations. The effectiveness of each method was quantified using metrics (PSNR, SSIM, and PCC), as described in the performance metrics section.

#### Spatially varying aberration estimation

To estimate spatially varying aberrations across a large field of view, our model was extended to accommodate local variations in wavefront distortions. We used the same pancreas sample and aberrating medium as in the sample-induced aberration correction experiments but expanded the field of view to 1094 μm × 1094 μm. This larger imaging area enabled the capture of regions exhibiting substantially different aberration characteristics. The phase-diversity acquisition protocol remained unchanged, with 22 images collected. In our spatially variant model, the image was divided into 8 × 8 patches, yielding 64 distinct regions. For each patch, a set of local aberration coefficients was estimated while simultaneously updating a common component. To prevent overfitting and encourage smooth spatial transitions, L1 regularization was applied to the local deviation terms. The regularization strength *β* was empirically chosen, providing sufficient constraint while allowing meaningful local variations to be captured. For comparison, we also processed the same dataset using a spatially invariant model that estimated only a single set of aberration coefficients for the entire field of view. This comparison enabled quantitative assessment of the improvements achieved by the spatially variant approach, particularly in regions where local aberrations deviated significantly from the common mode component. We applied an L1 regularization on the spatially varying component Δ*c*_*k*,*i*_: $L_{reg}=\beta \sum_{i}\left|\Delta c_{k,i}\right|$ where *β* is a hyperparameter that controls the strength of the constraint. This regularization term is added to the original loss function.

#### Ground truth image acquisition

For wide-field imaging experiments, a ground truth image for estimate object and aberration-corrected image was obtained by acquiring an image of the same pancreas tissue slide after removing the aberrating medium using a spinning disk confocal microscope (Andor Dragonfly 200, Oxford Instruments) equipped with a 10×, 0.3 NA water dipping objective lens (UMPLFLN10XW, Olympus). Confocal images were used as structural references because confocal microscopy provides optical sectioning and reduced out-of-focus background, thereby offering a higher-resolution representation of the underlying sample structure that more closely reflects the intrinsic object. All images were acquired with an exposure time of 1.00 s. The ground truth confocal image (0.61 μm/pixel) was resampled to match the pixel size of wide-field image (0.53 μm/pixel). For quantitative evaluation, we performed image registration between the wide-field images and the resampled ground truth confocal image.

#### Aberration correction in light-sheet microscopy of larval zebrafish brain

To evaluate the capability of GRAPHYCS on dynamic samples, we performed light-sheet imaging experiments of larval zebrafish brains expressing pan-neuronal nuclear localized GCaMP6s at imaging depths of 200 μm–300μm. To introduce additional known aberrations, the deformable mirror was controlled to modulate the wavefront using the defocus (Z_5_) mode. We implemented a triangular driving signal for phase-diversity acquisition, which was designed to acquire the images with no applied defocus (Z_5_ = 0 μm) multiple times, obtaining 22 images by continuously sweeping defocus (*Z*_5_) mode coefficient from −0.39 μmto +0.39 μm in equal steps with a field of view of 547 μm × 684 μm (1024 × 1280 pixels). This defocus range and number of diversity images were empirically determined to be effective for accurate aberration estimation and object estimation. All images were acquired with an exposure time of 1.00 s. Although the exposure time was 1.00 s per frame, additional time was required for deformable mirror actuation and settling between frames. As a result, the total acquisition time for one complete phase-diversity sequence (22 images) was approximately 90 s under our experimental conditions. The aberration estimation process began by initializing the object estimate with the first aberrated image, the temporally varying component with the frame-wise differences between each image in the 22 diversity images and the first aberrated image, and setting all Zernike coefficients to zero. After estimating the aberration coefficients, we applied the corresponding corrective wavefront to the deformable mirror. This corrective wavefront was generated by inverting the phase of the estimated aberration. For comparison, we also processed the same dataset with a cropped region using the analytic PD method, and NeuWS for dynamic aberrations. In our spatially variant model, the field of view was divided into 10 × 8 patches, yielding 80 distinct regions. The optimization was performed by minimizing our defined loss function, as described in the training details and parameters. The effectiveness of each method was quantified using metrics (DCTS, and Fourier sharpness), as described in the performance metrics section. We applied an L1 regularization on the temporally varying component Δ*o*_*n*_(*x*,*y*): $L_{reg}=\eta \sum_{n=1}^{N}\Delta o_{n}\left(x,y\right)$ where *η* is a hyperparameter controlling the strength of the constraint. Neurons were manually segmented by drawing regions of interest (ROIs) on the temporal maximum intensity projection of the estimated time-varying object, and activity traces were extracted from these ROIs

#### Sample preparation for wide-field microscopy imaging

For all imaging experiments, we used commercial microscope sample slides of the lymph node (Microscope slides, DIY-SCIENCE) and pancreas (Biological specimen, USCAMEL). To create the aberrating medium, a mouse brain section was placed on a glass-bottom Petri dish and covered with a coverslip. 2% agar solution containing 50% w/v sucrose was placed on this coverslip. Another coverslip was placed on top of the agar layer. The mouse brain section had a thickness of approximately 50 μm, and the total thickness of the aberrating medium (including brain section, agarose with sucrose, and coverslips) was approximately 1.50 mm, well within the working distance of our objective lens (3.5 mm). The commercial tissue slides were positioned beneath this aberrating medium for sample-induced aberration experiments. For system aberration correction experiments and PSF measurements, samples were imaged directly without the aberrating medium. For zebrafish experiments, we used transgenic larval zebrafish expressing calcium indicator pan-neuronal nuclear localized GCaMP6s with a Casper (mitfa(w2/w2);mpv17(a9/a9)) and nacre (mitfa(w2/w2)) mutant were imaged at 4–6 days post fertilization. This indicator was controlled by the neuron-specific HuC promoter, resulting in the transgenic line Tg(HuC:H2B-GCaMP6s).^46^^,^^47^ The larvae were paralyzed by bath incubation with 0.25 mg/mL of pancuronium bromide (Sigma-Aldrich) solution for 2 min.^48^ After paralysis, the larvae were embedded in agar using a 2% low-melting point agarose (Thermo Scientific) on a specimen holder which is mounted on a custom sample chamber. The custom chamber was filled with standard fish water after solidifying the agarose gel.

#### Training details and parameters

For the spatially invariant forward model, the estimated object *o*, the Zernike coefficients of the phase aberration *c*_*k*_, and the self-calibration parameters *θ* and *γ*_*k*_ are found using the following optimization process:

$$\;{o^{*},c}_{k}^{*},\theta^{*},\gamma_{k}^{*}=\underset{o,c_{k}\theta ,\gamma_{k}}{\arg\;\;\min}\sum_{n=1}^{N}\left|I_{n}\left(x,y\right)-q_{n}\left(x,y\right)\right|+\beta \left|F\left\{I_{n}\left(x,y\right)\right\}-F\left\{q_{n}\left(x,y\right)\right\}\right|\text{.}$$

For the spatially variant forward model, the spatially varying coefficients were added to the set of parameters estimated and an L1 penalty term was added to prevent excessively spatially variant aberration estimations:

$${o^{*},c}_{k}^{*},{\;\Delta c_{k,i}^{*},\theta }^{*},\gamma_{k}^{*}=\underset{{o,c}_{k},\Delta c_{k,i},\theta ,\gamma_{k}}{\arg\;\;\min}\sum_{n=1}^{N}\left|I_{n}\left(x,y\right)-q_{n}\left(x,y\right)\right|+\alpha \left|F\left\{I_{n}\left(x,y\right)\right\}-F\left\{q_{n}\left(x,y\right)\right\}\right|+\beta \sum_{i}\left|\Delta c_{k,i}\right|\text{.}$$

For the spatially invariant forward model to handle dynamic samples, the estimated temporally varying component Δ*o*_*n*_ and the motion parameters *ρ*_*n*_ were added to the estimated parameters and an L1 penalty term was added to prevent excessive temporally varying component estimation:

$$o_{B}^{*}{,{\Delta o}_{n}^{*},c}_{k}^{*},\rho_{n}^{*},\theta^{*},\gamma_{k}^{*}=\underset{o_{B},\Delta o_{n},c_{k},\rho_{n},\theta ,\gamma_{k}}{\arg\;\;\min}\sum_{n=1}^{N}\left|I_{n}\left(x,y\right)-q_{n}\left(x,y\right)\right|+\alpha \left|F\left\{I_{n}\left(x,y\right)\right\}-F\left\{q_{n}\left(x,y\right)\right\}\right|+\eta \sum_{n=1}^{N}\Delta o_{n}\left(x,y\right)\text{.}$$

For the spatially variant forward model to handle dynamic samples, the spatially varying coefficients were further added to the set of estimated parameters and an L1 penalty term was again added to prevent excessively spatially variant aberration estimations:

$${o_{B}^{*},{\Delta o}_{n}^{*},c}_{k}^{*},\Delta c_{k,i}^{*},\rho_{n}^{*},\theta^{*},\gamma_{k}^{*}=\underset{o_{B},\Delta o_{n},c_{k},\Delta c_{k,i},\rho_{n},\theta ,\gamma_{k}}{\arg\;\;\min}\sum_{n=1}^{N}\left|I_{n}\left(x,y\right)-q_{n}\left(x,y\right)\right|+\alpha \left|F\left\{I_{n}\left(x,y\right)\right\}-F\left\{q_{n}\left(x,y\right)\right\}\right|+\eta \sum_{n=1}^{N}\Delta o_{n}\left(x,y\right)+\beta \sum_{i}\left|\Delta c_{k,i}\right|\text{.}$$

We implemented all computations using PyTorch^49^ 2.2.1 on NVIDIA RTX 2080 GPUs. Computation time and peak GPU memory consumption reported in Table S3 were measured on an NVIDIA RTX 5090 GPU. The optimization was conducted using the Adam optimizer^50^ across 2000, 1000, and 500 training epochs for simulated data, wide-field imaging data, and light-sheet imaging data, respectively. A comprehensive sensitivity analysis of key hyperparameters is presented in Figure S19; Table S5 and S6.

The aberration coefficient learning rate was set to 1 × 10^−2^ for simulated data, 5 × 10^−3^ for widefield data using the spatially invariant model, 1 × 10^−2^ for wide-field data using the spatially variant model, and 5 × 10^−3^ for light-sheet data. The learning rate on the object parameters was set to 2 × 10^−3^ for simulated data, 5 × 10^−3^ when using the spatially invariant model on widefield and light-sheet data, 2 × 10^−3^ when using the spatially variant model on widefield data, 5 × 10^−3^ when using the spatially invariant model and 5 × 10^−4^ when using the spatially variant model on light-sheet data The learning rate on the affine transformation parameters was set to 1 × 10^−6^ for simulated data without system imperfections, 1 × 10^−3^ for simulated data in the presence of system imperfections, 5 × 10^−3^ when using the spatially invariant model on widefield data, and 1 × 10^−4^ when using the spatially variant model on wide-field data as well as on light-sheet data. The learning rate on the scale parameters was set equal to the learning rate of the affine transformation parameters except in the case of simulated data with system imperfections, where it was reduced to 5 × 10^−4^. For light-sheet data, the learning rates on the temporally varying component and motion parameters were set to 1 × 10^−3^ and 1 × 10^−5^, respectively. Loss weights were set to 1.0 for the L1 image domain loss and *α* = 7 × 10^−4^, 5 × 10^−4^, 1 × 10^−3^ for the L1 Fourier domain loss on simulated data, wide-field data using the spatially invariant forward model, and on wide-field and light-sheet data using the spatially variant forward model, respectively. The weight on the L1 regularization on spatially varying coefficients was set to *β* = 2 × 10^−3^ for wide-field data and *β* = 1 × 10^−3^ for light-sheet data. The weight on the L1 regularization on the temporally varying component for light-sheet data was set to *η* = 1.

#### Performance metrics

To quantitatively evaluate the performance of GRAPHYCS, we employed root-mean-square (RMS) wavefront distortion (or wavefront RMS error), peak signal-to-noise ratio (PSNR), structural similarity index measure (SSIM), and Pearson’s correlation coefficient (PCC) for simulated data. For experimental data, we used PSNR, SSIM, and PCC when ground truth images were available (such as system aberration corrected images or confocal reference images). When ground truth was unavailable, we utilized a set of reference-free image quality metrics: a Fourier transform-based sharpness metric,^27^ and the normalized discrete cosine transform Shannon entropy (DCTS).^42^ First, we calculated the wavefront RMS error, defined as the root-mean-square difference between the estimated wavefront phase Φ_*Estimated*_ and the ground truth wavefront phase Φ_*Groundtruth*_ according to $\sqrt{\frac{1}{N}\sum_{n=1}^{N}{\left(\Phi_{Ground\;truth,i}-\Phi_{Estimated,i}\right)}^{2}}$, where *N* is the number of sampling points across the pupil. The PSNR between the image *I*_1_ and the reference image *I*_2_ is defined as PSNR(*I*_1_,*I*_2_) = $10\cdot \log_{10}\left(\frac{R^{2}}{MSE}\right)$ where *R* is the range of the pixel values in the reference image and the mean square error (MSE) is defined as MSE = $\frac{1}{N}\sum_{n=1}^{N}{\;\left(I_{1,n}-I_{2,n}\right)}^{2}\;\text{.}$ The SSIM between the image *I*_1_ and the reference image *I*_2_ is defined as SSIM(*I*_1_,*I*_2_) $=\frac{\left(2\mu_{1}\mu_{2}+C_{1}\right)\left(2\sigma_{1,2}+C_{2}\right)}{\left(\mu_{1}^{2}+\mu_{2}^{2}+C_{1}\right)\left(\sigma_{1}^{2}+\sigma_{2}^{2}+C_{2}\right)}$ where *μ*_1_ and *μ*_2_ are the mean values of *I*_1_ and *I*_2_, *σ*_1_ and *σ*_2_ are the standard deviations of image *I*_1_ and *I*_2_, *σ*_1,2_ is the covariance between *I*_1_ and *I*_2_, and *C*_1_ and *C*_2_ are stabilization constants that prevent division by zero. The Pearson’s correlation (PCC) between the image *I*_1_ and the reference image *I*_2_ is defined as PCC = $\frac{\sum_{n=1}^{N}\left(I_{1,n}-\mu_{1}\right)\left(I_{2,n}-\mu_{2}\right)}{\sqrt{\sum_{n=1}^{N}{\left(I_{1,n}-\mu_{1}\right)}^{2}}\sqrt{{\sum_{n=1}^{N}\left(I_{2,n}-\mu_{2}\right)}^{2}}}$ where *μ*_1_ and *μ*_2_ are the mean values of *I*_1_ and *I*_2_, and *I*_1,*n*_ and *I*_2,*n*_ are the *n*-th pixel values. The Fourier transform-based sharpness metric is the ratio of higher to lower spatial frequency content in the image, which depends on aberration content but remains independent of overall brightness changes, and is defined as Fourier sharpness metric = $\frac{\sum_{\eta f_{\max}<\left|f\right|<\;f_{\max}}|F\left\{I\right\}|}{\sum_{0<\left|f\right|<\eta f_{\max}}|F\left\{I\right\}|}$ where |F{I}| is the absolute value of the Fourier transform of the image, $f_{\max}=\frac{2NA}{\lambda }$, and *η* is a scaling factor. This Fourier sharpness metric was calculated following ref.^27^ The scaling factor *η*was set to 0.2 in our implementation. This ratio effectively captures the sharpness of the PSF and the restoration of fine details in the corrected images. The DCTS of the image was calculated following ref.^42^ To mitigate the influence of border artifacts on performance metrics, all evaluations of the estimated object were performed after cropping to remove the edges prior to computing PSNR, SSIM, PCC, Fourier sharpness, and DCTS.

#### PSF measurement

To quantify the improvement in imaging performance, we characterized the point spread function (PSF) of our optical system both before and after system aberration correction. For PSF measurements, we used yellow-green fluorescent beads with a diameter of 500 nm (15700-10, Polysciences), which approximate point sources given our system’s resolution limit. The fluorescent bead samples were diluted in distilled water at a ratio of 1:10,000 to ensure adequate isolation of individual beads, and were prepared on a microscope glass slide. The measured PSF was then fitted with a Gaussian function to obtain the full width at half maximum (FWHM) values. The FWHM values were calculated using the estimated standard deviation of the fitted Gaussian function.

#### Integration of illumination profile into forward model

To account for non-uniform illumination for wide-field imaging, we integrated the illumination profile into the image formation model (Figure S20). The illumination profile was obtained by averaging 20 images of a fluorescent slide (FSK2, Thorlabs) acquired with a field of view of 1094 μm × 1094 μm, followed by Gaussian smoothing with σ = 17 μm (corresponding to 32 pixels). The resulting image was normalized to the [0, 1] intensity range using its maximum value. This illumination profile, matched to the field of view used for image acquisition, was incorporated into the forward model for all experiments presented in Figures 3 and 4.

#### Comparison with other algorithms

We used the publicly available implementation of analytic PD (https://github.com/ceej640/PhaseDiversity), and NeuWS (https://github.com/Intelligent-Sensing/NeuWS). For analytic PD, we used the same number of Zernike modes (12 modes from primary astigmatism to tetrafoil) for aberration parameterization. For NeuWS, the estimated wavefront aberration was projected on the basis spanned by the same number of Zernike modes (12 modes from primary astigmatism to tetrafoil) using least squares projection for aberration parameterization.

### Quantification and statistical analysis

All quantitative performance evaluations and statistical analyses were performed using MATLAB. Detailed information on image quality metrics and wavefront estimation accuracy, including the exact value of n, what n represents (e.g., number of synthetic datasets with different underlying wavefront aberrations under the same non-ideal conditions), and dispersion measures (e.g., mean ± standard deviation), can be found in the respective figure legends and tables. Computation time and peak GPU memory consumption were evaluated using custom Python software.
