# Supplementary material for: Graph-based modeling of optical system enables adaptive optics on dynamic samples with self-calibration
Source: iScience. 2026 Jul 14;29(8):116769. doi: 10.1016/j.isci.2026.116769 (PMC13382438; doi:10.1016/j.isci.2026.116769)
Supplement: Document S1. Figures S1–S20 and Tables S1–S6 [file mmc1.pdf]

iScience, Volume *29*

Supplemental information

Graph-based modeling of optical system enables adaptive optics on dynamic samples with self-calibration

Eun-Seo Cho, Joon Park, Hyungwon Jin, Yoonjae Chung, Minho Eom, Hyejin Shin, Jae-Byum Chang, Jung-Hoon Park, and Young-Gyu Yoon

SUPPLEMENTARY FIGURES

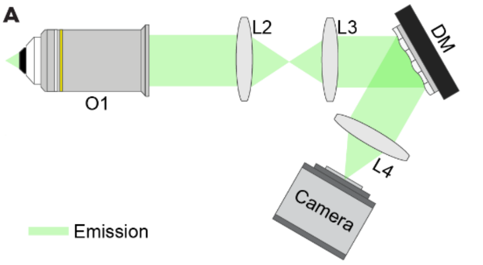

B

Component	Description
O1	10X 0.3 NA objective (f = 20mm)
L2	Achromatic lens (f = 180mm)
L3	Achromatic lens (f = 150mm)
M1	Fold mirror
M2	Fold mirror
DM	Deformable mirror surface
L4	Tube lens (f = 200mm)

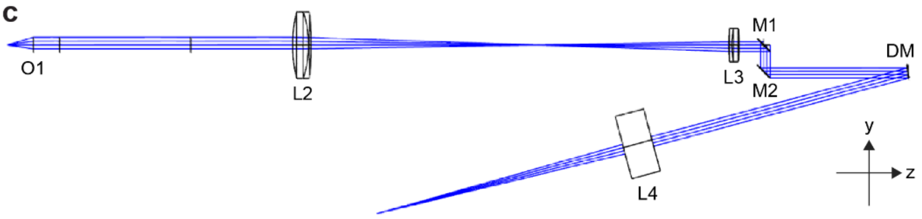

Figure S1. Optical model of the emission path in wide-field adaptive optics system.

(A) Schematic of the emission path in wide-field adaptive optics system. The diagram illustrates the optical layout from the object through the objective lens, relay lenses, deformable mirror (DM), and tube lens to the camera.

(B) List of optical components in the optical simulation software.

(C) Optical model of the emission path implemented in the optical simulation software using the components in **(B)**.

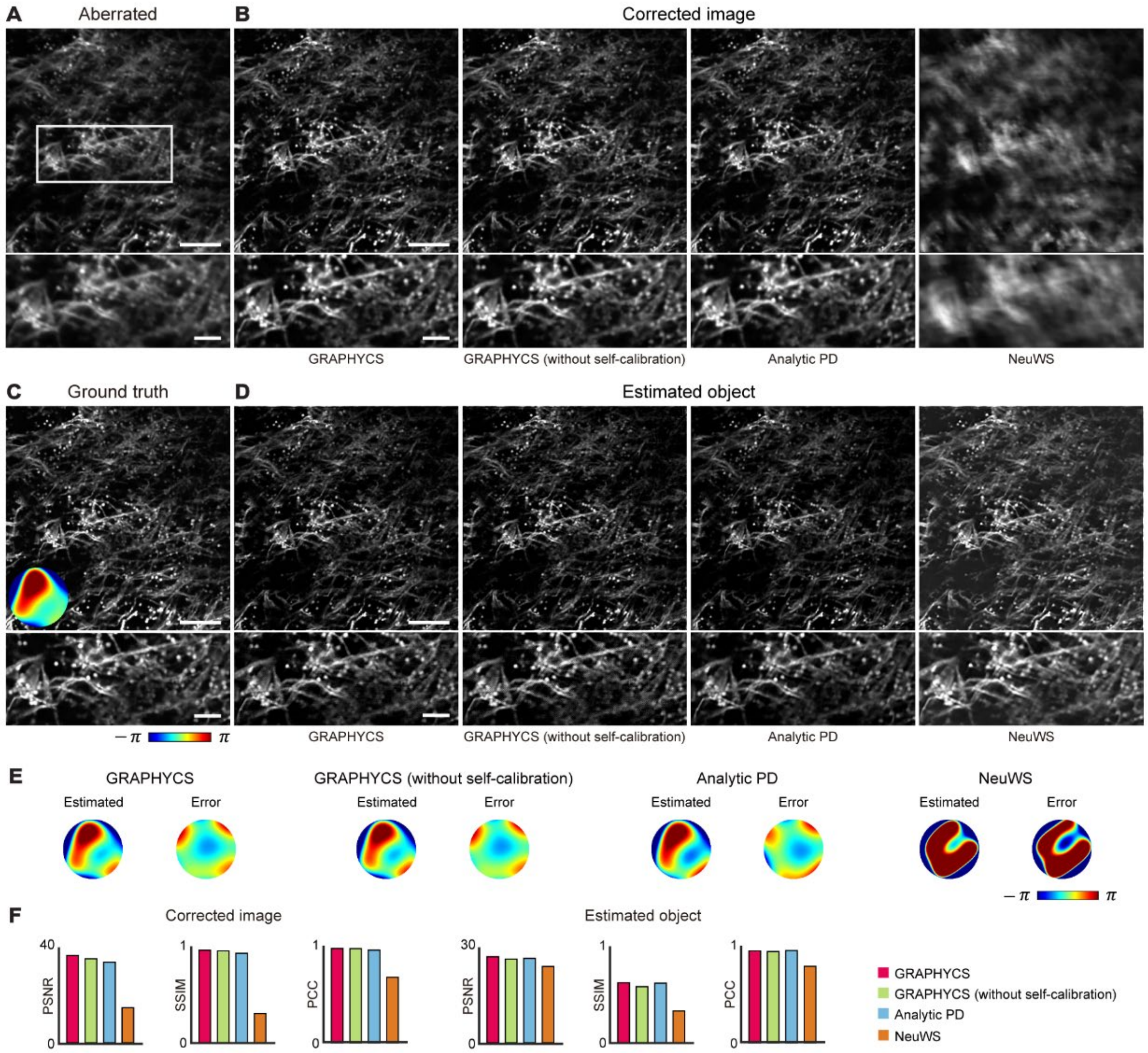

Figure S2. Performance validation on simulated data under ideal conditions.

(A) Aberrated image simulated without system non-idealities. Scale bar, 50 μm. A magnified view of the boxed region is presented below. Scale bar, 20 μm.

(B) Aberration-corrected images of GRAPHYCS, GRAPHYCS without self-calibration, analytic PD, and NeuWS (from left to right). Scale bar, 50 μm. A magnified view of the boxed region in **(A)** is presented below. Scale bar, 20 μm.

(C) Ground truth object image. Scale bar, 50 μm. A magnified view of the boxed region in **(A)** is presented below. Scale bar, 20 μm.

(D) Estimated object images of GRAPHYCS, GRAPHYCS without self-calibration, analytic PD, and NeuWS (from left to right). Scale bar, 50 μm. A magnified view of the boxed region in **(A)** is presented below. Scale bar, 20 μm.

(E) Estimated wavefront aberrations and corresponding wavefront error maps from GRAPHYCS, GRAPHYCS without self-calibration, analytic PD, and NeuWS (from left to right).

(F) Quantitative comparison of image quality metrics (PSNR, SSIM, and PCC) for the aberration-corrected images (left) and the estimated object images (right) under ideal conditions.

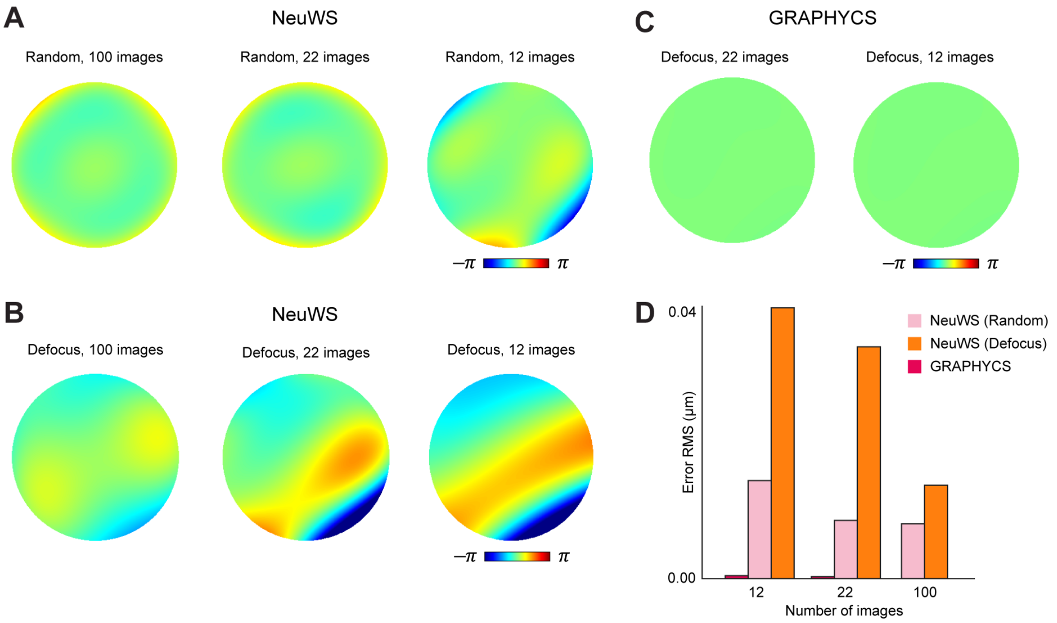

Figure S3. Performance evaluation of NeuWS and GRAPHYCS under different phase-diversity configurations.

(A) Aberration estimation results obtained using NeuWS with random phase diversity under different numbers of images (100, 22, and 12).

(B) Aberration estimation results obtained using NeuWS with defocus phase diversity under different numbers of images (100, 22, and 12).

(C) Aberration estimation results obtained using GRAPHYCS with defocus phase diversity using 22 and 12 images.

(D) Quantitative comparison of wavefront estimation error for NeuWS and GRAPHYCS under different phase-diversity configurations and numbers of images. The plotted values correspond to the measurements summarized in Table S1.

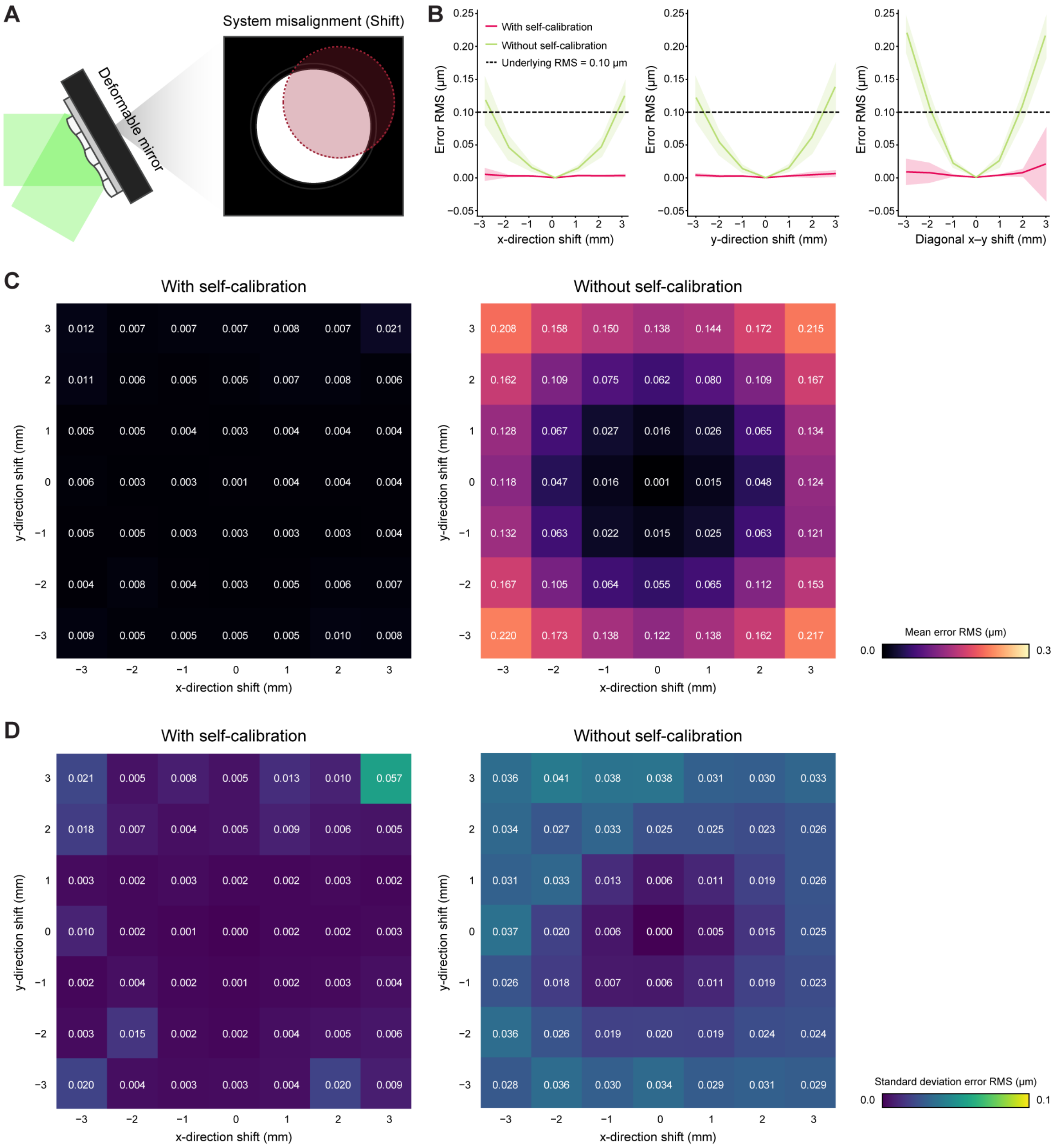

Figure S4. Aberration estimation performance with and without self-calibration under increasing system misalignment.

(A) Schematic of simulated misalignment. The incident beam amplitude at the deformable mirror plane was shifted with shift magnitudes ranging from −3.0 to +3.0 mm in 1.0 mm increments.

(B) Mean wavefront RMS error as a function of beam shift magnitude along the x-direction (left), y-direction (middle), and diagonal x–y direction (right). Mean values are shown as solid lines, and the shaded areas indicate the mean ± standard deviation calculated from 20 underlying wavefront aberrations with an RMS magnitude of 0.10 µm. The dashed line indicates the RMS magnitude of the underlying wavefront aberration used for simulation.

(C) Mean wavefront RMS error across lateral beam shifts in the x and y directions displayed as heatmaps for GRAPHYCS with self-calibration (left) and without self-calibration (right).

(D) Standard deviation of wavefront RMS error across lateral beam shifts in the x and y directions displayed as heatmaps for GRAPHYCS with self-calibration (left) and without self-calibration (right).

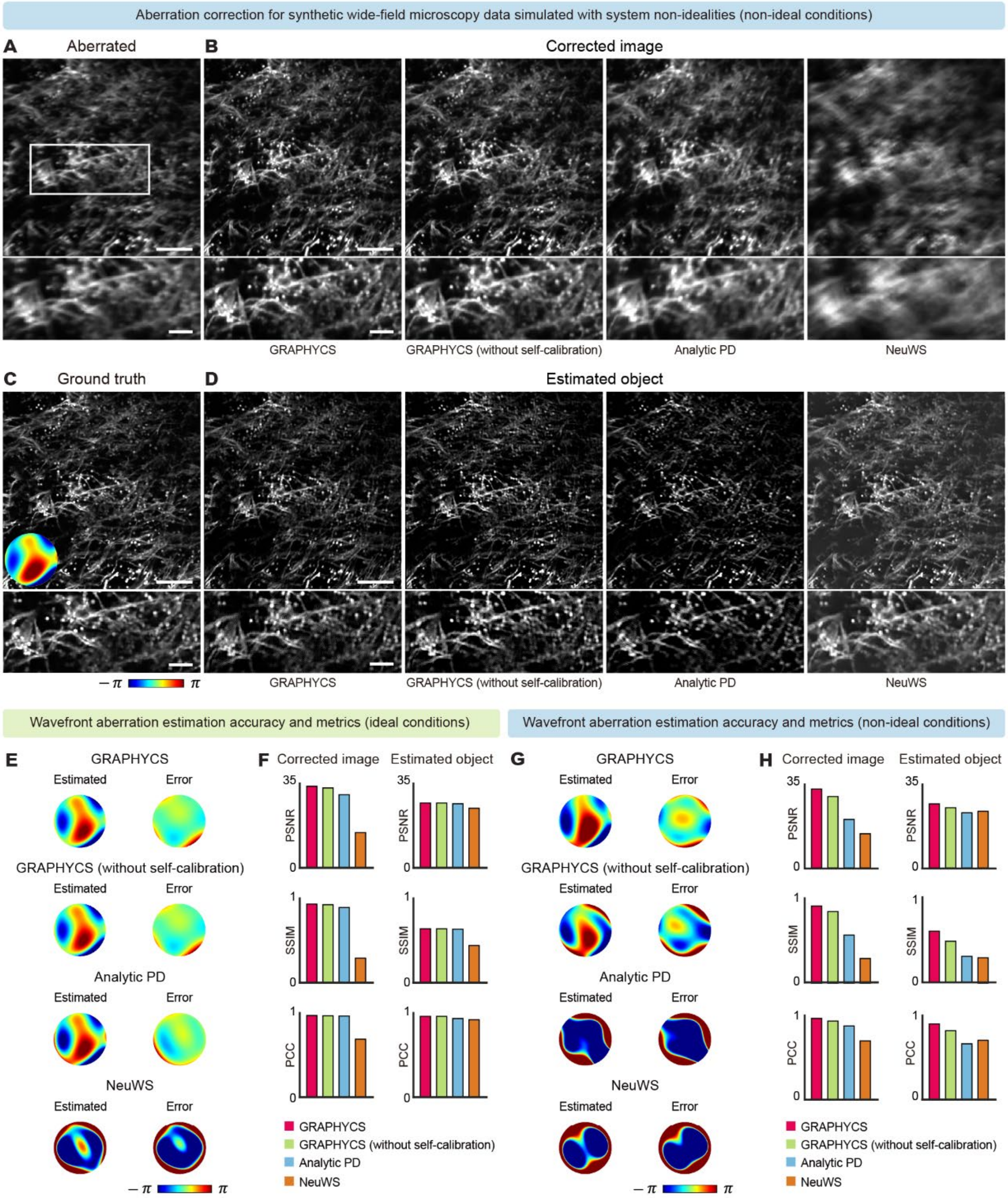

Figure S5. Performance validation on simulated data.

(A) Aberrated image simulated under system non-idealities introduced by controlled misalignments to a deformable mirror, including a lateral shift of −2.5 mm in x and +1.5 mm in y and an additional 1.0-degree tilt. Scale bar, 50μm. A magnified view of the boxed region is presented below. Scale bar, 20 μm

(B) Aberration-corrected images of GRAPHYCS, GRAPHYCS without self-calibration, analytic PD, and NeuWS (from left to right). Scale bar, 50 μm. A magnified view of the boxed region in **(A)** is presented below. Scale bar,

20 μm.

(C) Ground truth object image along with ground truth wavefront aberrations. Scale bar, 50 μm. A magnified view of the boxed region in **(A)** is presented below. Scale bar, 20 μm.

(D) Estimated object images of GRAPHYCS, GRAPHYCS without self-calibration, analytic PD, and NeuWS (from left to right). Scale bar, 50 μm. A magnified view of the boxed region in **(A)** is presented below. Scale bar, 20 μm.

(E) Estimated wavefront aberrations (left column) and corresponding wavefront error maps (right column) from GRAPHYCS, GRAPHYCS without self-calibration, analytic PD, and NeuWS (top to bottom).

(F) Quantitative comparison of image quality metrics (PSNR, SSIM, and PCC) for the aberration-corrected images (left) and the estimated object images (right) under ideal conditions.

(G) Estimated wavefront aberrations (left column) and corresponding wavefront error maps (right column) from GRAPHYCS, GRAPHYCS without self-calibration, analytic PD, and NeuWS (top to bottom).

(H) Quantitative comparison of image quality metrics (PSNR, SSIM, and PCC) for the aberration-corrected images (left) and the estimated object images (right) under non-ideal conditions.

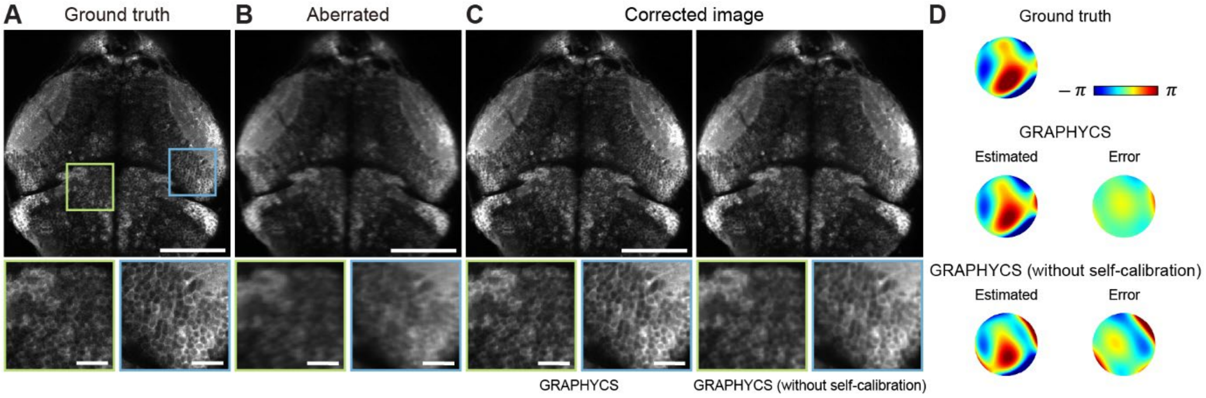

Figure S6. Performance validation on simulated data under non-ideal conditions.

(A) Ground truth object image of a larval zebrafish brain. Scale bar, 100 μm. Magnified views of the boxed regions are presented below. Scale bar, 20 μm.

(B) Aberrated image simulated under system non-idealities introduced by controlled misalignments to a deformable mirror, including a lateral shift of −2.0 mm in x and +1.0 mm in y and an additional 1.0-degree tilt. Scale bar, 100 μm. Magnified views of the boxed regions in **(A)** are presented below. Scale bar, 20 μm.

(C) Aberration-corrected images of GRAPHYCS (left) and GRAPHYCS without self-calibration (right). Scale bar, 100 μm. Magnified views of the boxed regions in **(A)** are presented below. Scale bar, 20 μm.

(D) Ground truth wavefront aberration (top), and the estimated wavefront aberrations (left column) with their corresponding wavefront error maps (right column) from GRAPHYCS and GRAPHYCS without self-calibration (middle and bottom).

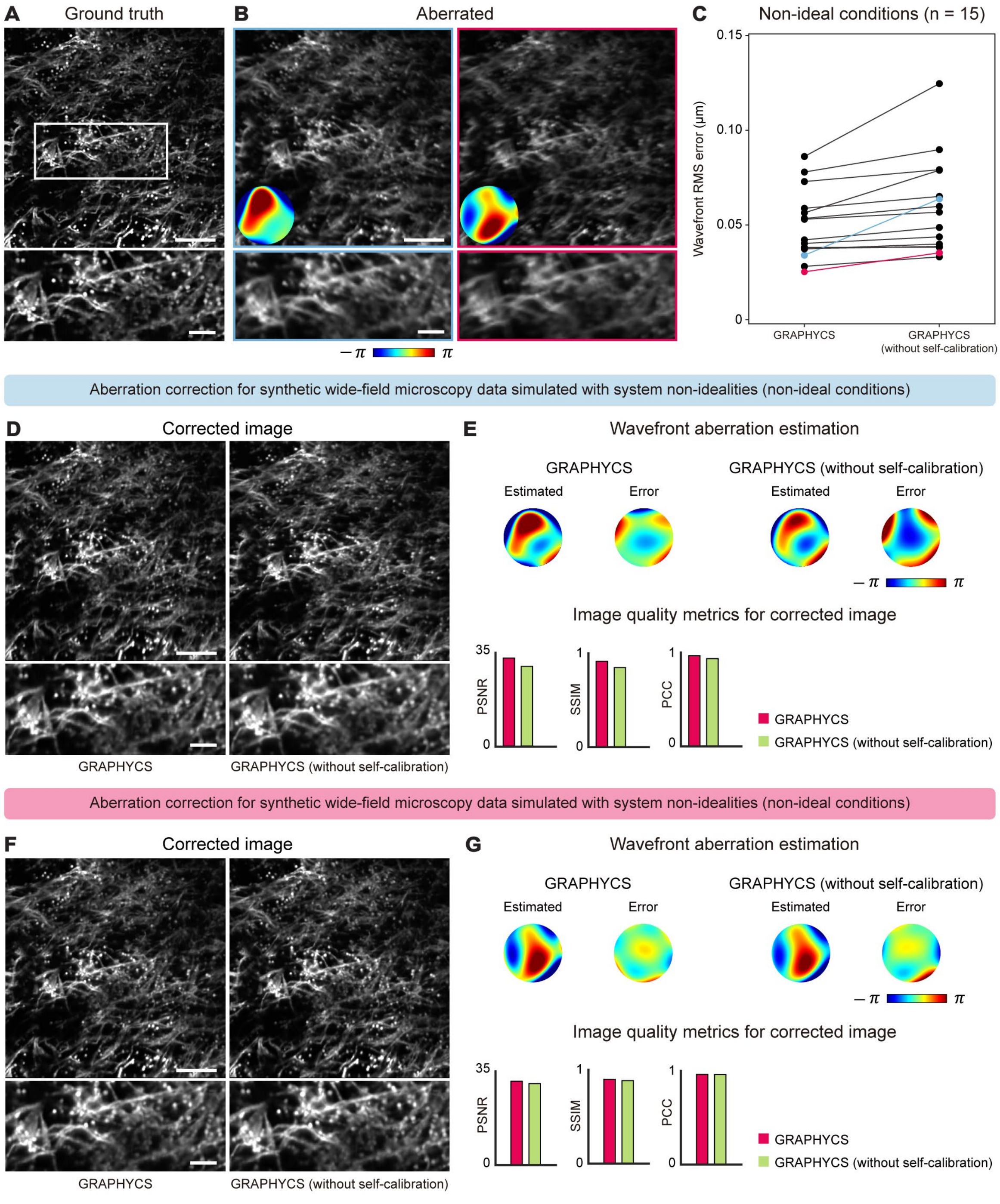

Figure S7. Comparison of two simulations with different underlying aberrations under same non-ideal condition.

(A) Ground truth object image. Scale bar, 50 µm. A magnified view of the boxed region is presented below. Scale bar, 20 µm.

(B) Aberrated image simulated under system non-idealities introduced by controlled misalignments to a deformable mirror, including a lateral shift of −2.0 mm in x and +1.0 mm in y and an additional 1.0-degree tilt, along with the corresponding ground truth wavefront aberration. Scale bar, 50 µm. A magnified view of the boxed

region in **(A)** is shown below. Scale bar, 20 μm.

(C) Paired comparison of wavefront RMS error between GRAPHYCS and GRAPHYCS without self-calibration across simulations performed under same non-ideal conditions (n = 15). Colored dot-connected lines correspond to the representative cases shown in **(B)**.

(D) Aberration-corrected images of GRAPHYCS (left) and GRAPHYCS without self-calibration (right) for the blue-highlighted representative case shown in **(B)**. Scale bar, 50 μm. A magnified view of the boxed region in **(A)** is presented below. Scale bar, 20 μm.

(E) Estimated wavefront aberrations (left) and corresponding wavefront error maps (right) obtained by GRAPHYCS and GRAPHYCS without self-calibration for the blue-highlighted representative case shown in **(B)**. Quantitative comparison of image quality metrics (PSNR, SSIM, and PCC) for the aberration-corrected images is shown below.

(F) Aberration-corrected images of GRAPHYCS (left) and GRAPHYCS without self-calibration (right) for the red-highlighted representative case shown in **(B)**. Scale bar, 50 μm. A magnified view of the boxed region in **(A)** is presented below. Scale bar, 20 μm.

(G) Estimated wavefront aberrations (left) and corresponding wavefront error maps (right) obtained by GRAPHYCS and GRAPHYCS without self-calibration for the red-highlighted representative case shown in **(B)**. Quantitative comparison of image quality metrics (PSNR, SSIM, and PCC) for the aberration-corrected images is shown below.

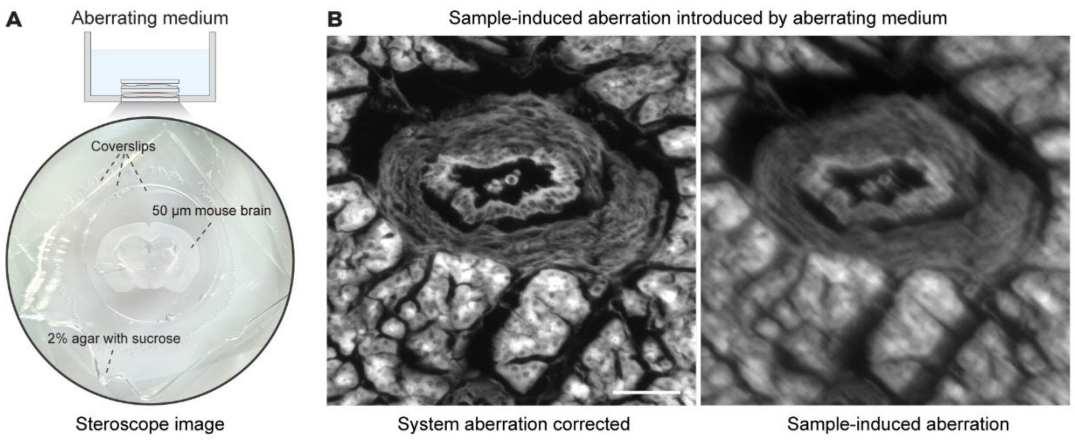

Figure S8. Sample-induced aberration using aberrating medium.

(A) Stereoscope image of the aberrating medium consisting of a 50 μm thick mouse brain slice, 2% agar containing 50% w/v sucrose, and coverslips.

(B) Sample-induced aberration introduced by the aberrating medium. System aberration-corrected image of pancreas tissue without the medium (left) and image of the same sample with the medium (right). Scale bar, 50 μm.

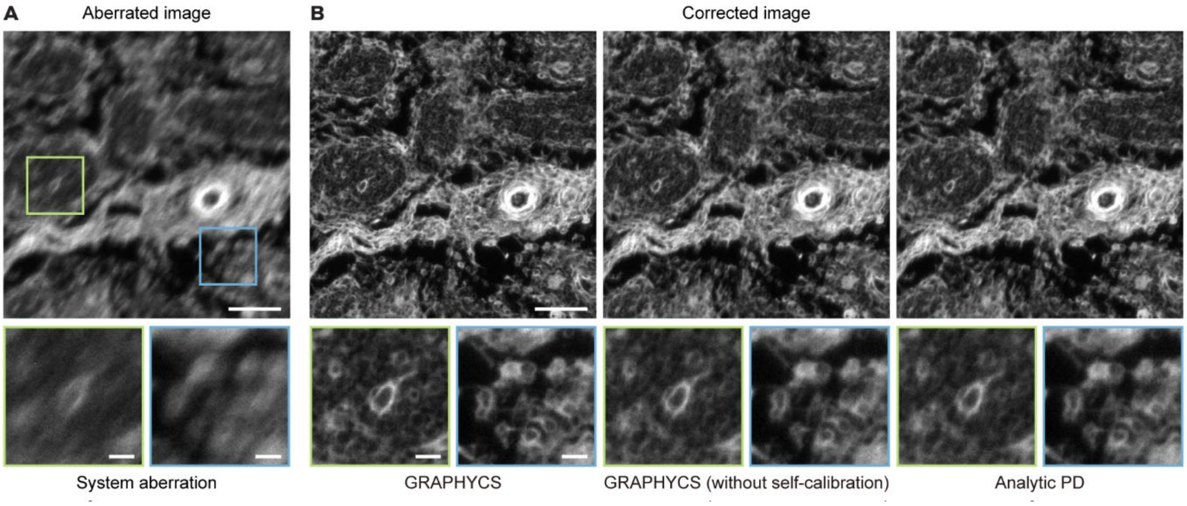

Figure S9. Correction of system aberration.

(A) Aberrated image of lymph node sample. Scale bar, 50 µm. Magnified views of the boxed regions are presented below. Scale bar, 10 µm.

(B) System aberration-corrected images of GRAPHYCS, GRAPHYCS without self-calibration, and analytic PD (from left to right). Scale bar, 50 µm. Magnified views of the boxed regions in **(A)** are presented below. Scale bar, 10 µm.

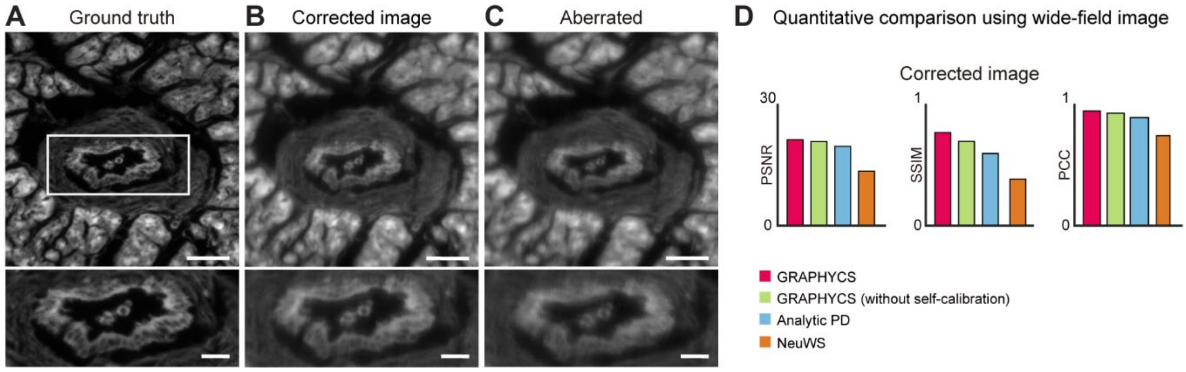

Figure S10. Validation of aberration correction using wide-field reference ground truth image.

(A) Ground truth image acquired with a wide-field microscope. Scale bar, 50 µm. A magnified view of the boxed region is presented below. Scale bar, 20 µm.

(B) Aberration-corrected image. Scale bar, 50 µm. A magnified view of the boxed region in **(A)** is presented below. Scale bar, 20 µm.

(C) Aberrated image. Scale bar, 50 µm. A magnified view of the boxed region in **(A)** is presented below. Scale bar, 20 µm.

(D) Quantitative comparison of image quality metrics (PSNR, SSIM, and PCC) evaluating the aberration-corrected image against the wide-field ground truth.

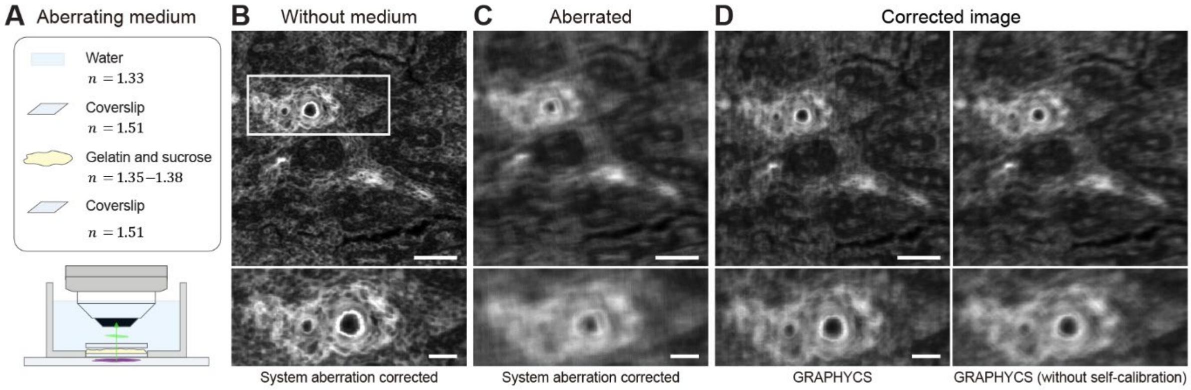

Figure S11. Correction of aberrations introduced by refractive index mismatch.

(A) Schematic of the experimental setup for introducing aberrations by refractive index (RI) mismatch. The aberrating medium consisted of a gelatin and sucrose layer ($n = 1.35 - 1.38$) sandwiched between coverslips ($n = 1.51$), placed in a glass-bottom Petri dish with the sample slide positioned below, introducing refractive index mismatches with the surrounding water ($n = 1.33$).

(B) Wide-field microscopy image of a lymph node sample after system aberration correction acquired without the medium. Scale bar, 50 µm. Magnified views of the boxed region are presented below. Scale bar, 20 µm.

(C) Aberration introduced by RI mismatch. Aberrated image with the medium acquired prior to sample-induced aberration correction. Scale bar, 50 µm. Magnified views of the boxed region in **(B)** are presented below. Scale bar, 20 µm.

(D) Aberration-corrected images using GRAPHYCS (left) and GRAPHYCS without self-calibration (right). Scale bar, 50 µm. Magnified views of the boxed region in **(B)** are presented below. Scale bar, 20 µm.

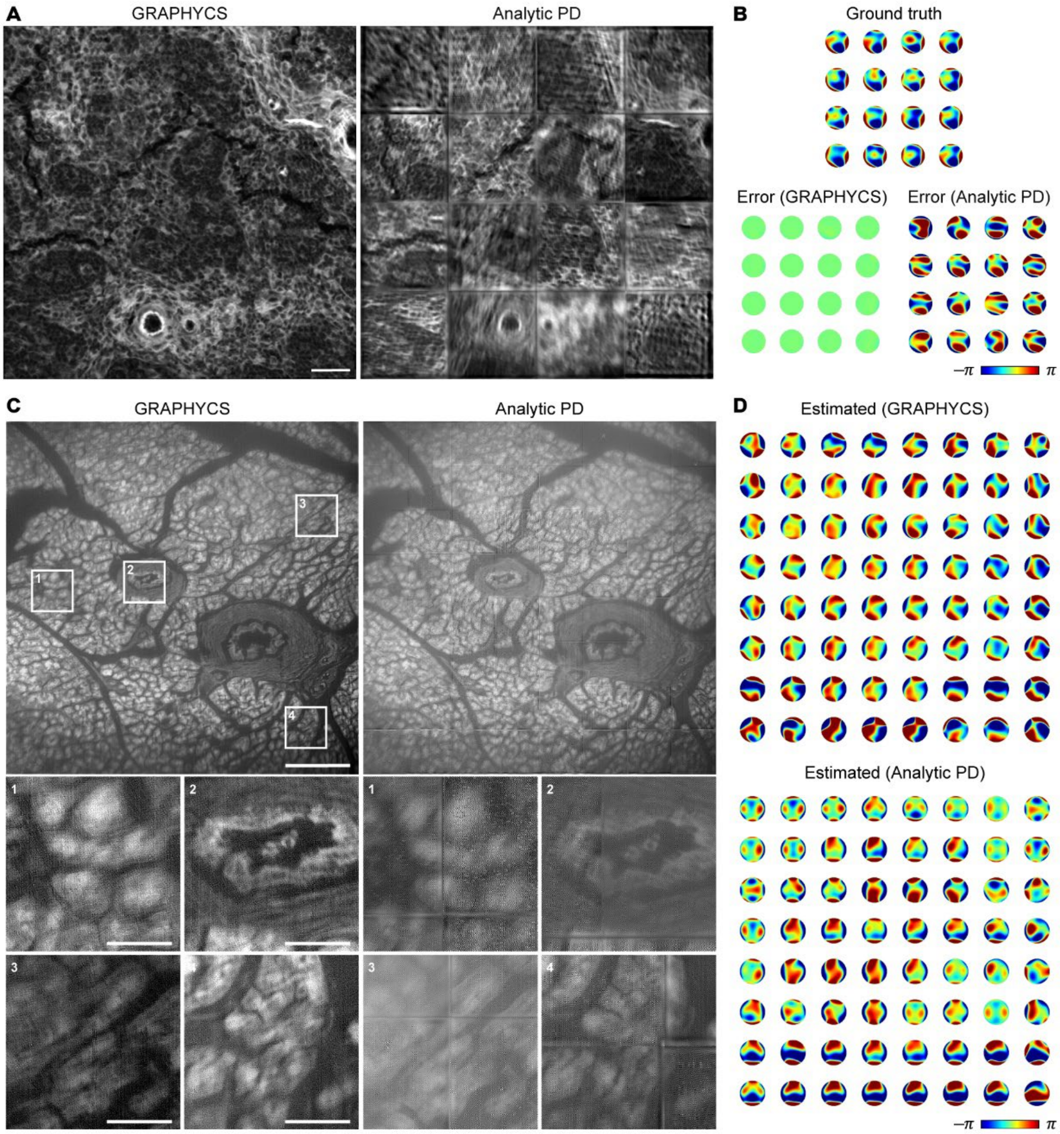

Figure S12. Comparison of GRAPHYCS and patch-wise analytic PD.

(A) Estimated object images from GRAPHYCS (left), and concatenated patch-wise analytic PD results (right). Scale bar, 50 µm.

(B) Ground truth spatially varying wavefront aberrations used for simulation (top), and corresponding wavefront error maps for GRAPHYCS (left bottom) and analytic PD (right bottom), shown patch-wise.

(C) Object estimation results on wide-field data of a pancreas sample with a field of view of 1094 µm × 1094 µm using GRAPHYCS (left) and concatenated patch-wise analytic PD results (right). Scale bar, 200 µm. Magnified views of the boxed regions are presented below. Scale bar, 50 µm.

(D) Spatially varying wavefront aberration estimations from GRAPHYCS (top) and analytic PD (bottom), shown patch-wise across the full field of view.

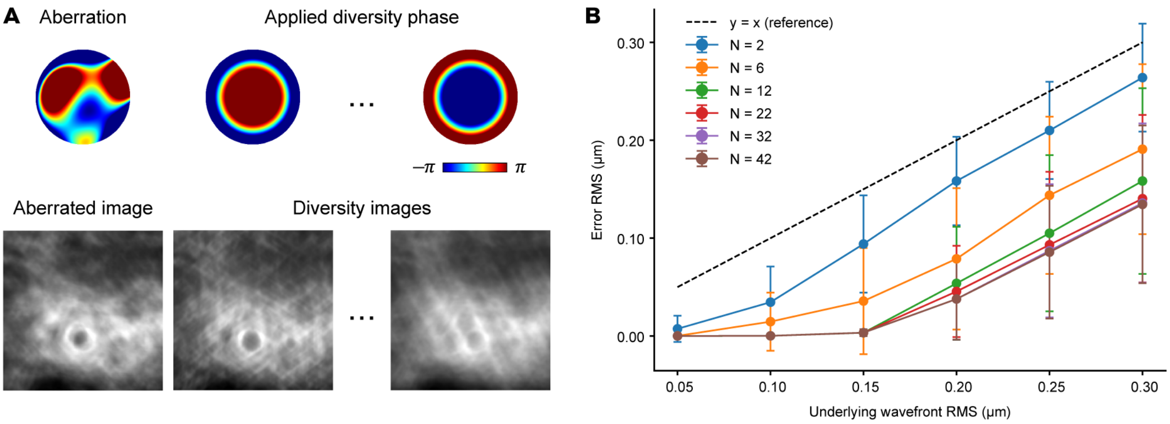

Figure S13. Comparison of the number of phase-diversity images on aberration estimation performance.

(A) Phase-diversity simulation approach. An underlying wavefront aberration was used to simulate an aberrated image, and defocus (Z5) was applied to generate phase-diversity images.

(B) Wavefront RMS error for different magnitudes of underlying RMS wavefront aberrations when applying defocus as a diversity phase at 0.20 μm RMS. Error bars indicate mean ± standard deviation over 10 random underlying wavefront aberrations.

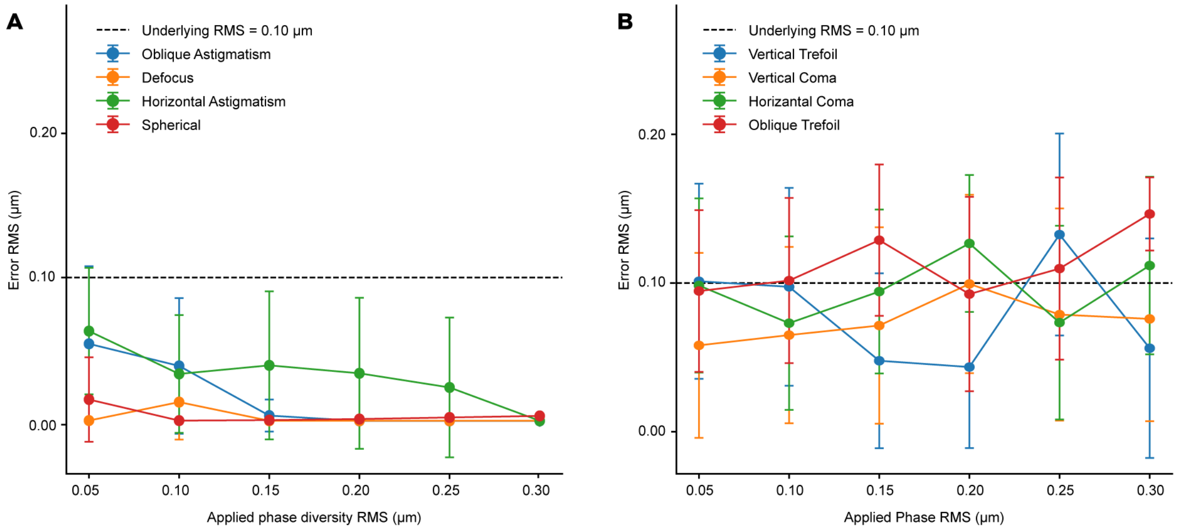

Figure S14. Comparison of diversity phase modulation strategies.

(A) Wavefront RMS error as a function of the RMS amplitude of the applied diversity phase for oblique astigmatism (Z4), defocus (Z5), vertical astigmatism (Z6), and spherical aberration (Z13). The dashed line indicates the underlying RMS wavefront aberration used for simulation (0.10 μm). Error bars indicate mean ± standard deviation over 10 random underlying wavefront aberrations.

(B) Wavefront RMS error as a function of the RMS amplitude of the applied diversity phase for vertical trefoil (Z7), vertical coma (Z8), horizontal coma (Z9), and oblique trefoil (Z10). The dashed line indicates the underlying RMS wavefront aberration used for simulation (0.10 μm). Error bars indicate mean ± standard deviation over 10 random underlying wavefront aberrations.

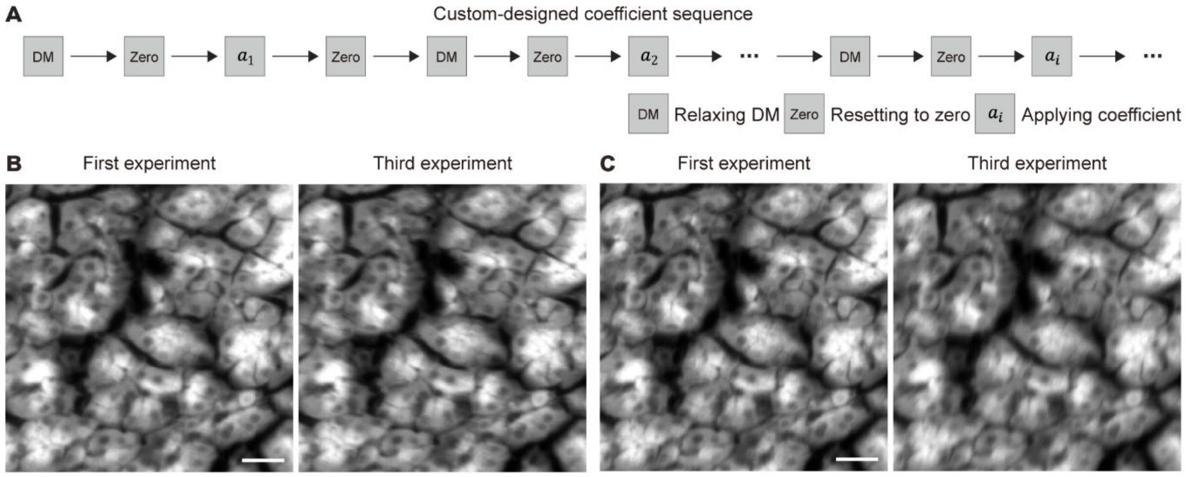

Figure S15. Custom-designed coefficient sequence for phase-diversity image acquisition.

(A) Schematic of custom-designed coefficient sequence. The sequence comprises three key components: (1) relaxing the deformable mirror (DM) using the Relax function in the DM control software to minimize hysteresis effects by applying a damped voltage oscillation around the original value; (2) resetting to zero by returning all Zernike coefficients to 0, which serves as the consistent reference value, with a voltage of 100 V applied to all segment electrodes of the DM; and (3) applying coefficient a_i for phase-diversity acquisition, where $i = 1,2,\cdots,N$ and N is the number of images acquired in the sequence.

(B) Experimental results with the sequence. Images of the pancreas tissue from the first (left) and the third (right) experiments, acquired at the reference point ($Z_5 = 0$ μm) after sweeping the Z_5 Zernike mode (defocus) from −3.25 μm to +3.25 μm in equal steps. Scale bar, 20 μm.

(C) Experimental results without the sequence. Images of the same sample from the first (left) and the third (right) experiment at the same reference point after identical coefficient sweeps. Scale bar, 20 μm.

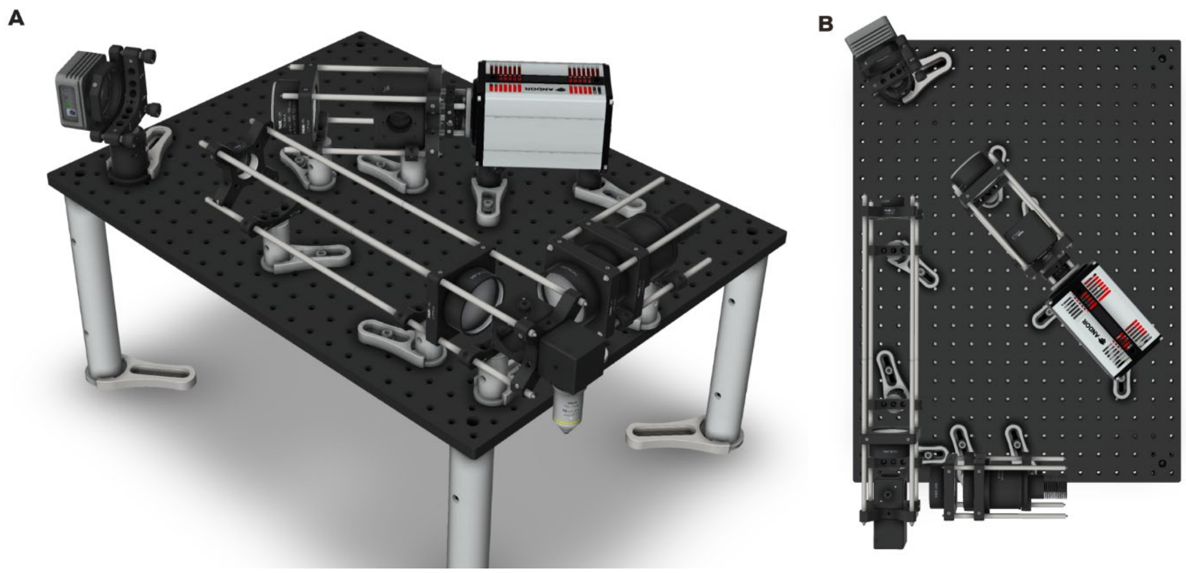

Figure S16. 3D rendering of wide-field adaptive optics system implementation.

(A) Tilted view of the optical setup implementation.

(B) Top view of the optical setup implementation.

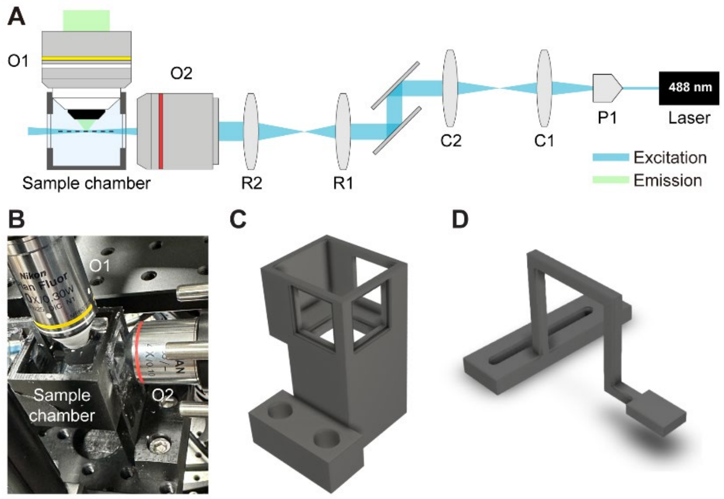

Figure S17. Implementation of the light-sheet illumination setup.

(A) Schematic of a light-sheet illumination setup in a light-sheet adaptive optics system.

(B) Photograph of a light-sheet imaging setup. The sample chamber was sealed with coverslips.

(C) 3D rendering of custom sample chamber.

(D) 3D rendering of custom specimen holder.

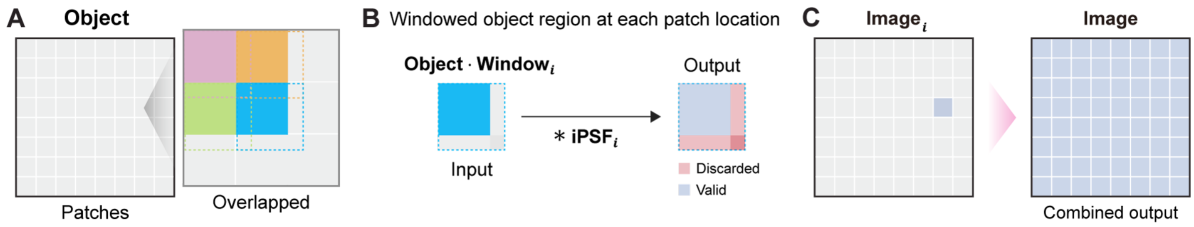

Figure S18. Overlap-save method for spatially varying image formation model.

(A) The field of view is divided into patches to account for spatially varying wavefront distortions across regions (left), where a representative 3×3 region illustrates the overlapping windows of neighboring patches indicated by dashed boxes (right).

(B) Each windowed object region as an input image is convolved with the spatially varying intensity point spread function (iPSF), and the overlapping regions in the convolution output image are discarded, keeping only the artifact-free valid region.

(C) The valid region of each patch forms an individual output image, which is placed at its corresponding position (left). All individual output images are concatenated to form the final combined output image (right).

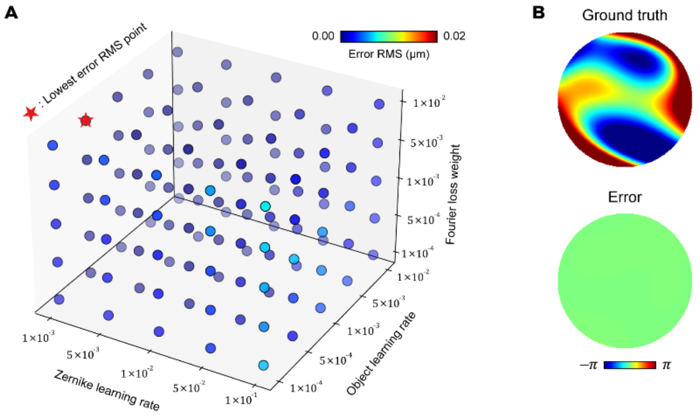

Figure S19. Parameter sensitivity analysis for spatially invariant aberration estimation.

(A) Sensitivity analysis of the optimization hyperparameters, including the learning rates for the Zernike coefficients and the object parameters, and the Fourier loss weight. Each point corresponds to a tested parameter combination, with color indicating the resulting wavefront RMS error. The red star denotes the parameter set that achieved the lowest wavefront error.

(B) Ground truth wavefront aberrations used for simulation (top), and corresponding lowest wavefront error map (bottom).

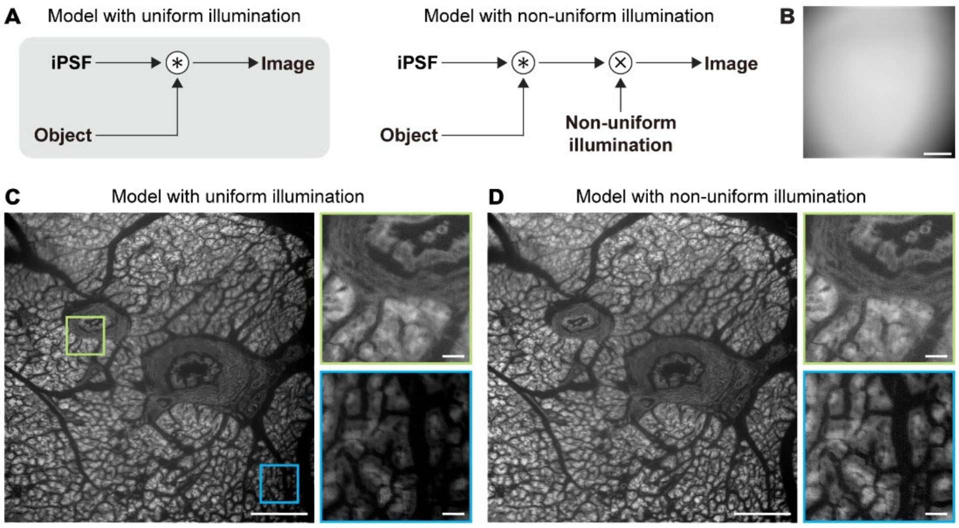

Figure S20. Image formation model incorporating non-uniform illumination.

(A) Schematic of image formation models assuming uniform illumination (left) and incorporating non-uniform illumination profile (right).

(B) Non-uniform illumination profile obtained by averaging 20 images of the fluorescent slide with a field of view of 1094 μm × 1094 μm, followed by Gaussian smoothing ($\sigma = 17$ μm) and normalization to the [0, 1] intensity range. Scale bar, 200 μm.

(C) Estimated object using spatially variant model with uniform illumination. Scale bar, 200 μm. Magnified views of the boxed regions are presented to the right. Scale bar, 20 μm.

(D) Estimated object using spatially variant model with non-uniform illumination. Scale bar, 200 μm. Magnified views of the boxed regions in **c** are presented to the right. Scale bar, 20 μm.

SUPPLEMENTARY TABLE

Table S1. Wavefront errors for NeuWS and GRAPHYCS across different phase diversity configurations.

Phase diversity	Number of images	Wavefront error RMS (μm)
Random (NeuWS)	100	0.0080
Random (NeuWS)	22	0.0086
Random (NeuWS)	12	0.0144
Defocus (NeuWS)	100	0.0137
Defocus (NeuWS)	22	0.0340
Defocus (NeuWS)	12	0.0398
Defocus (GRAPHYCS)	22	0.0003
Defocus (GRAPHYCS)	12	0.0005

Table S2. Quantitative comparison on simulated data under non-ideal conditions.

Non-ideal conditions		Aberration-corrected image			Estimated object		
Method	Wavefront RMS error (μm)	PSNR (dB)	SSIM	PCC	PSNR (dB)	SSIM	PCC
GRAPHYCS	0.0252	30.76	0.8890	0.9616	27.67	0.6857	0.9410
GRAPHYCS (without self-calibration)	0.0353	29.93	0.8859	0.9590	26.99	0.6408	0.9211
Analytic PD	0.2367	20.42	0.5819	0.8888	23.68	0.3945	0.7488
NeuWS	0.2213	17.40	0.4309	0.8039	23.99	0.3463	0.7799

Table S3. Computation time and peak GPU memory consumption.

Experiment	Method	Data size	Time (sec)	GPU (GB)
Simulation (Figure 2)	GRAPHYCS	512×512×12	79.87	0.365
	Analytic PD	512×512×12	1.92	2.722
	NeuWS	512×512×12	234.96	1.691
Wide-field (Figure 3)	GRAPHYCS	512×512×22	43.46	0.637
	Analytic PD	512×512×22	73.97	3.072

	NeuWS	512×512×22	419.53	1.702
Wide-field (Figure 4)	GRAPHYCS (invariant)	2048×2048×22	510.45	7.159
	GRAPHYCS (variant)	2048×2048×22	1049.81	6.798
	Analytic PD	2048×2048×22	1232.93	22.541
	NeuWS	2048×2048×22	10661.30	10.301
Light-sheet (Figure 5C, E)	GRAPHYCS (dynamic)	1024×1024×22	91.43	2.372
	Analytic PD	1024×1024×22	351.69	10.758
	NeuWS (dynamic)	1024×1024×22	2354.63	8.309
Light-sheet (Figure 5F)	GRAPHYCS (dynamic, variant)	1280×1024×22	279.75	9.303
	Analytic PD	1280×1024×22	717.68	20.877
	NeuWS (dynamic)	1280×1024×22	3909.99	12.977

Table S4. Effect of image crop size on computational memory consumption and training time.

Data size	Time (sec)	GPU (GB)	Convergence Time* (sec)	Wavefront error RMS (μm)
128×128×22	20.73	0.146	9.46	0.0019
256×256×22	25.20	0.245	9.64	0.0006
512×512×22	36.84	0.600	8.85	0.0010
1024×1024×22	139.82	1.933	41.69	0.0008

* Time taken for the wavefront error RMS to decrease below λ / 50

Table S5. Parameter sensitivity test for spatially variant aberrations for static samples.

L1 regularization weight ($\boldsymbol{\beta}$)	Wavefront error RMS (μm)	PCC	SSIM	PSNR
1×10^{-4}	0.0007	0.9943	0.9828	34.38
2×10^{-4}	0.0007	0.9942	0.9828	34.39
5×10^{-4}	0.0007	0.9943	0.9829	34.40
1×10^{-3}	0.0007	0.9943	0.9829	34.40

Table S6. Parameter sensitivity test for spatially invariant aberrations for dynamic samples.

Motion parameter	Temporal L1 regularization weight ($\boldsymbol{\eta}$)	Wavefront RMS error (μm)	PCC	SSIM	PSNR

1×10^{-3}	1×10^{-1}	0.0008	0.9387	0.8593	30.52
1×10^{-3}	2×10^{-1}	0.0008	0.9489	0.8423	29.76
1×10^{-3}	5×10^{-1}	0.0006	0.9481	0.8253	29.22
1×10^{-3}	1	0.0009	0.9341	0.8192	29.07
1×10^{-4}	1×10^{-1}	0.0008	0.9570	0.8714	30.91
1×10^{-4}	2×10^{-1}	0.0008	0.9645	0.8539	30.03
1×10^{-4}	5×10^{-1}	0.0012	0.9556	0.8346	29.43
1×10^{-4}	1	0.0007	0.9511	0.8311	29.29
1×10^{-5}	1×10^{-1}	0.0010	0.9712	0.8881	31.38
1×10^{-5}	2×10^{-1}	0.0007	0.9656	0.8525	29.98
1×10^{-5}	5×10^{-1}	0.0008	0.9554	0.8315	29.35
1×10^{-5}	1	0.0007	0.9497	0.8298	29.26
